# A smooth vetch (*Vicia villosa* var.) strain endogenous to the broad-spectrum antagonist *Bacillus siamensis* JSZ06 alleviates banana wilt disease

**DOI:** 10.3389/fpls.2024.1410197

**Published:** 2024-06-04

**Authors:** Yan-Nan Ruan, Caihong Nong, Attachai Jintrawet, Huacai Fan, Libo Fu, Si-Jun Zheng, Shu Li, Zhi-Yuan Wang

**Affiliations:** ^1^ Institute of Agricultural Environment and Resources, Yunnan Academy of Agricultural Sciences, Kunming, Yunnan, China; ^2^ College of Agronomy and Life Sciences, Kunming Universities, Kunming, Yunnan, China; ^3^ Faculty of Agriculture, Chiang Mai University, Chiang Mai, Thailand

**Keywords:** smooth vetch, *Bacillus siamensis*, *Fusarium* wilt of banana, mechanism of inhibition, pot experiment, LC-MS/MS, biological control

## Abstract

*Fusarium* wilt, caused by *Fusarium oxysporum* f. sp. *cubense* Tropical Race 4 (*Foc* TR4), poses a significant threat to banana production globally, thereby necessitating effective biocontrol methods to manage this devastating disease. This study investigates the potential of *Bacillus siamensis* strain JSZ06, isolated from smooth vetch, as a biocontrol agent against *Foc* TR4. To this end, we conducted a series of *in vitro* and *in vivo* experiments to evaluate the antifungal activity of strain JSZ06 and its crude extracts. Additionally, genomic analyses were performed to identify antibiotic synthesis genes, while metabolomic profiling was conducted to characterize bioactive compounds. The results demonstrated that strain JSZ06 exhibited strong inhibitory activity against *Foc* TR4, significantly reducing mycelial growth and spore germination. Moreover, scanning and transmission electron microscopy revealed substantial ultrastructural damage to *Foc* TR4 mycelia treated with JSZ06 extracts. Genomic analysis identified several antibiotic synthesis genes, and metabolomic profiling revealed numerous antifungal metabolites. Furthermore, in pot trials, the application of JSZ06 fermentation broth significantly enhanced banana plant growth and reduced disease severity, achieving biocontrol efficiencies of 76.71% and 79.25% for leaves and pseudostems, respectively. In conclusion, *Bacillus siamensis* JSZ06 is a promising biocontrol agent against *Fusarium* wilt in bananas, with its dual action of direct antifungal activity and plant growth promotion underscoring its potential for integrated disease management strategies.

## 1 Introduction

Banana (*Musa* spp.) stands as a prominent tropical and subtropical fruit crop worldwide. It holds the distinction of being one of the most produced, traded, and consumed fruits globally (FAO, 2023). Following rice, wheat, and maize, bananas have ascended to become the fourth major food crop in developing nations (Aguilar-Hawod et al., 2019; Zhou et al., 2023). China holds the position of the largest global importer and the second-largest manufacturer of bananas. The economic progress of China’s tropical regions is significantly dependent on the banana industry (Zheng et al., 2018). *Fusarium* wilt of banana, induced by *Fusarium oxysporum* f. sp. *cubense* (*Foc*), poses substantial threats to production by diminishing yields in terms of quantity and quality. This can result in regional and global fluctuations within the banana industry (Ghag et al., 2015; FAO, 2023). *Foc* pathogens are categorized into four pathogenic microspecies, namely *Foc* race 1, 2, 3, and 4, based on variations in banana infestation (Zhou et al., 2023). In particular, the TR4 – a variant of tropical race 4 – possesses the capability to infect a wide range of banana genotypes combinations susceptible to TR4 infection (Zhou et al., 2023). Currently, it is spreading swiftly across all global banana regions, posing a threat to approximately 80% of banana plants due to *Foc* TR4 (Damodaran et al., 2019; García-Bastidas et al., 2020).

*Fusarium oxysporum* f. sp. *cubense* Tropical Race 4 (*Foc* TR4) is a soil-borne pathogen that rapidly disseminates through the transportation by susceptible plants, soil, water, agricultural tools, and other human activities (Swarupa et al., 2014; Zhang et al., 2021a). The spores of the pathogen can persist in the soil for decades, and currently, there is no established effective method for controlling *Foc* TR4 (Ghag et al., 2015; Ploetz, 2015). At present, strategies for controlling banana wilt primarily revolve around the development and breeding of new disease-resistant varieties, as well as agricultural, physical, chemical, and biological control methods, etc (Davis and Grant, 1996; Ploetz, 2015; Shen et al., 2019). Chemical control stands out as the most effective approach among these strategies; however, it contributes to environmental pollution and other issues (Latz et al., 2016; He et al., 2021). On the other hand, agricultural and physical management is intricate and challenging to execute (Bubici et al., 2019). Moreover, considering the triploid nature of bananas and the associated difficulty in cultivating disease-resistant varieties (Zhou et al., 2023), this contributes to the constraint in *Foc* TR4 control. Conversely, in light of the growing emphasis on sustainable agricultural development, beneficial microorganisms are currently acknowledged as one of the most promising approaches for *Foc* TR4 control, characterized by safety, environmental friendliness, and efficiency (He et al., 2021; Yun et al., 2022).

During the last decades, *Bacillus* spp. have been extensively used as biocontrol agents (Bubici et al., 2019; He et al., 2021). In particular, the genus *Bacillus* has long been recognized as an ideal biological control agent due to the abundance and stability of its metabolites and its ease of cultivation (Fan et al., 2021; Li et al., 2021a). Studies have shown that soil administration of *Bacillus subtilis* increases the diversity of soil microorganisms, stimulates native antagonistic flora, and enhances tolerance to the propagation of banana wilt (Gadhave et al., 2018; Tao et al., 2020). *Bacillus endophyticus* may cause systemic stress in crops through the production of cyclic lipopeptides or the jasmonone/ethylene signaling cascade (van Loon, 2007; Sun et al., 2022). Some *Bacillus* species produce antibiotics through the Non-Ribosomal Peptide Synthase (NRPS) and Polyketide Synthase (PKS) genes, for example, *Bacillus velezensis* produces surfactin, *Bacillus amyloliquefaciens* contains bacillomycin D, and fungitoxin is effective in antagonizing banana wilt (Xu et al., 2013; Li et al., 2021a). Furthermore, certain *Bacillus* strains promote banana growth by producing IAA and other volatile compounds after colonising the plant (Idris et al., 2007; Tan et al., 2013). However, these *Bacillus* species’ antimicrobial activity can still be affected by their complex growth environment and different pathogenic fungi (Saravanan et al., 2003). Thus, it is necessary to identify and isolate *B.* spp. with antagonistic activity against various plant pathogenic fungi.

Many scholars have widely isolated endophytic *Bacillus cenocepaci* from banana plants (Souza et al., 2014) or their inter-root soils (Zhang et al., 2011), and these endophytic bacteria tend to show advantages of bioprophylaxis and colonization (Xue et al., 2015). However, it is possible that efficient antagonistic bacteria still exist in other plant materials. Some studies have reported the isolation of potent antagonists from weeds, medicinal plants, and chili peppers (Adler, 1980; Zeng-Hui et al., 2007). Endophytic *Burkholderia cenocepacia* 869T2 isolated from the roots of *Vanilla planifolia* showed good biocontrol efficacy in a field trial (86%) (Ho et al., 2015). Endophytic strains of *Serratia marcescens* ITBB isolated from rubber trees B5-1 also showed high inhibitory activity in the greenhouse (79%) and in the field (70%) (Tan et al., 2015). Two strains of endophytic nitrogen fixers – namely *Burkholderia* spp. and *Herbaspirillum*-like – isolated from pineapple roots and stems have also been reported promising candidate strains for biocontrol and biofertilizer of banana, and both of them were isolated from plants other than banana (Weber et al., 2007). Therefore, banana endophytic antagonists are widely present in various plant species, and the isolation of efficient and green endophytic antagonists is still a research direction for banana biocontrol. Smooth vetch (*Vicia villosa* var.) is an annual legume that can enhance soil quality, reduce use of mineral fertiliser and improve plant resistance, which is essential for ensuring high plant yields and creating a good agro-ecological environment (Gao et al., 2023; Liang et al., 2023). Earlier research has shown that planting smooth vetch can recruit large quantities of soil plant growth regulating rhizobacteria (PGPR) (Xiao et al., 2017; Shi et al., 2022), Some of the PGPR can colonize into the interior of smooth vetch to generate dominant strains, which can play the function of promote plant growth and resisting diseases, thus improving crop yield and resistance (Abeywickrama et al., 2023; Pu et al., 2023). Other studies have found that intercropping bananas with green manure reduces the *Fusarium spinosum* population and thus substantially lowers the prevalence of banana wilt (Yang et al., 2022a). It is possible that smooth vetch harbors a large number of endophytic bacteria with good disease resistance-promoting properties (Yang et al., 2022b; Liang et al., 2023). Thus, smooth vetch can be used as a good material for the isolation of biocontrol-promoting bacteria.

A new strain of *Bacillus siamensi*s JSZ06, isolated from the stem of smooth vetch, was subjected to taxonomic classification based on 16S rRNA gene sequencing, supplemented by morphological, physiological, and whole genome data. Preliminary studies evaluated its *in vitro* inhibitory effect on *Foc* TR4 and its *in vivo* biocontrol and growth-promoting effects on potted plants. Additionally, the fungicidal activity of the fermentation broth extract was assessed, focusing on its impact on *Foc* TR4 mycelium and spores, and elucidating its bactericidal mechanism. Chemical composition analysis was performed using liquid chromatography-mass spectrometry (LC-MS). This study aims to provide scientific evidence supporting the potential of *B*. *siamensi*s JSZ06 as an endophytic antagonist for the biological control of banana wilt disease.

## 2 Materials and methods

### 2.1 Smooth vetch plant sample collection

The smooth vetch plant samples (stem, leaf, root) were collected from Songming County, Kunming, Yunnan Province, China (102° 41’ E, 25° 28’ N). Plant material was kept in insulated containers at -80°C after being transferred to the lab in clean polyethylene bags. The banana variety was ‘Baxi’ (*Musa* spp. AAA), which was propagated by the Agricultural Biodiversity Research Laboratory of Yunnan Provincial Agriculture Academy.

### 2.2 Phytopathogenic fungi

The banana research team of the Institute of Agricultural Environment and Resources, Yunnan Academy of Agricultural Sciences, isolated, identified, and stored *Foc* TR4 strain 15-1 (NCBI Accession Number: SRX9739537) as the test banana pathogen, from Brazilian banana varieties grown in Xishuangbanna fields. The samples were preserved strains with high virulence and pathogenicity based on guidelines described in Zheng et al. (2018).

### 2.3 Isolation of plant endophytes

The isolation of endophytes was initially performed by rinsing the plant surface with tap water. The plant was then split into roots, stems and leaves. To sterilize the surface of the plant material, 10 g of washed plant material was weighed and soaked in 75% (v/v) alcohol for 2 minutes, followed by 1% HgCl for 2 minutes, then rinsed three times with sterile distilled water. Subsequently, the roots, stems or leaves were excised and thoroughly crushed with the addition of quartz sand (0.2 g) and sterilized in a saline solution (pH = 7.4). A grinding solution prepared in 10^-3^, 10^-4^, 10^-5^ and 10^-6^ concentration gradients was applied to Luria-Bertani solid medium (LB; tryptone 10 g/L, yeast powder 5 g/L, sodium chloride 10 g/L and 15 g/L agar). To verify the effectiveness of the surface sterilization, the water used for the final rinse of the plants was spread on LB agar plates. All procedures were replicated thrice. Petri dishes were then incubated for 72 hr at 30°C. Single colonies were purified on LB medium and stored at -80°C in 50% (v/v) glycerol (Christakis et al., 2021).

### 2.4 Preliminary screening of endophytic bacteria antagonistic to *Foc* TR4

Using a dual culture assay, we evaluated the antagonistic effect of screened endophytic bacteria against *Fusarium oxysporum* f. sp. *cubense* Tropical Race 4 (*Foc* TR4) (Wei et al., 2020). We utilized a perforator to cut *Foc* TR4 mycelial discs of 5 mm diameter from the periphery of actively growing *Foc* TR4 plates. These discs were then centrally placed in the centre of potato dextrose agar (PDA; 200 g/L potato powder, 20 g/L dextrose or 15 g/L agar). Subsequently, the selected strains were inoculated at four symmetrical points around the *Foc* TR4 fungal cake, at a distance of about 2.5 cm. As a control, we used *Foc* TR4 plates without inoculation of the isolated strains. All procedures were replicated thrice. The diameter of pathogenic bacteria was measured by the crossover method after 5-7 days of incubation at 28°C. Use **Equation 1** to calculate the rate of inhibition (GI) of TR4 growth in each treatment group:


(1) GI=[(C−T)]×100%

The diameters of fungal mycelial growth in the treated and control plates were denoted by T and C, respectively.

### 2.5 Secondary screening of endophytic bacteria antagonizing *Foc* TR4

The preparation of crude extracts with antimicrobial activity was performed according to the method previously reported by Li et al. (2021c). A small-scale fermentation process of endophytic bacteria initially screened for antifungal activity was carried out. The specific method involved culturing the antagonistic endophytes in LB liquid medium at 30°C and 180 rpm for three days. Subsequently, the culture was centrifuged at 13,000 rpm and filtered through a 0.22 μm sterilizing filter to obtain the fermentation broth. This fermentation broth was then mixed with an equal volume of ethyl acetate, and crude extracts were obtained using organic solvent extraction. The mixture was subjected to ultrasonication for 1 hr and shaking for 8 h. Ten extraction was repeated three times. Post-extraction, the organic solvent layer was concentrated using a rotary evaporator in a 40°C water bath, resulting in a precipitate. This precipitate was dissolved in methanol (AR) and dried in a fume hood for further use (Li et al., 2021b). In order to find the extract’s antifungal activity tests, the crude extract was evaluated by agar pore spreading technique for its inhibitory activity on mycelial growth (Li et al., 2021c). Initially, 10 mg of the extract were dissolved in 1 M Dimethyl sulfoxide (DMSO), achieving a concentration of 10% (v/v), and thereafter, it was sterilized using a 0.22 µm filter membrane. Subsequently, four 5 mm microwells were meticulously punched on PDA agar plates employing a sterile punch. Consequently, 50 μL of the sterilized extract solution was carefully aspirated into each well. A similar volume with DMSO 10% v/v without 10 mg of raw extract served as untreated control. Inoculate a 5 mm diameter TR4 cake into the center of the petri dish. The antimycotic effect was evaluated by measuring the growth of hyphae after 7 days of incubation at 28°C. All products of the replicated experiments were incubated to inoculate three petri dishes.

### 2.6 Biolog identification and morphological analysis of biocontrol strains

To gain more insight into the morphological characteristics of endophytic bacteria that most effectively antagonise *Foc* TR4, endophytic bacteria were cultured on different agar media commonly available in the laboratory, including LB, PDA, Reasoner’s 2A, Nutrient Agar, Czapek-Dox Agar, Tryptic Soy Agar and Gao’s No. 1. Transverse sections of bacterial strains on LB dishes were taken from 1 cm^2^ and processed for 3 h at 4°C using 2.5% glutaraldehyde in order to obtain scanning electron microscope (SEM) images of the strains.

The purified JSZ06 strain was sent to Guangdong Microbial Strain Collection Center (Guangdong, China) for physiological and biochemical identification. The identification system was GEN III MicroPlatesTM (Biolog, Hayward, CA, USA), which contained 49 carbon sources and 20 biochemical assays.

### 2.7 Genome sequencing of strain JSZ06

The isolated strains were cultured in Luria-Bertani (LB) broth for 24 h at 30°C and 200 rpm with shaking. Subsequently, bacterial DNA was extracted from 1 mL of the culture using the Qiagen DNA extraction kit, following the manufacturer’s instructions. The extracted DNA was then subjected to whole genome sequencing, which was performed by Shanghai Majorbio Bio-pharm Technology Co., Ltd. Bioinformatics analysis of the sequencing data was conducted using the Majorbio cloud platform (www.majorbio.com) (Ren et al., 2022).

### 2.8 Construction of phylogenetic tree

16S rRNA sequences were extracted from the genome of strain JSZ06. To find the homology sequences, the 16S rRNA isolate was checked against NCBI GenBank (https://www.ncbi.nlm.nih.gov/genbank/) database and EzBioCloud server (https://www.ezbiocloud.net/identify) (Qi et al., 2021). Phylogenetic trees were created with the MEGA 7.0 neighbor-joining algorithm (Wei et al., 2020), with the Bootstrap value set to 1000 and the remainder of the parameters at default setting.

### 2.9 Characterization of PGP traits of endophytes

Using the technique described by Sheng et al. (2008), isolates were evaluated for the production of Indole Acetic Acid (IAA). Endophyte culture inocula were added to LB medium that had been 500 mg/L tryptophan added, the samples were then cultured at 30°C for 72 h. The supernatant from the centrifugation of fermentation broth at 8,000 rpm was used to detect IAA.

The production of iron carriers by isolated bacteria was evaluated using techniques described by previous authors Luo et al. (2011). An equal amount of CAS solution was mixed with bacterial supernatant cultured in CDA liquid medium. After incubation in the dark at room temperature for 3 h, the absorptivity level at 630 nm was measured. Subsequently, the readings were contrasted with the control’s OD_630_ (λ/λ0).

In a modified Pikovskayas media enriched with tricalcium phosphate, the endophytes’ ability to solubilize minerals was measured (Wang et al., 2018). After incubation of the test strains in phospholysis medium for 72 h at 30°C, dissolved phosphate in the supernatant was determined by the molybdenum blue method. The strains were tested for their potassium capacity (Zhang and Kong, 2014). The test strains were incubated in medium containing potassium feldspar powder for 72 h, and the amount of effective potassium in the supernatant was determined by flame spectrophotometry.

Endophytic bacteria were inoculated in a basic medium containing 10 mL of Dworkin-Foster (DF) with 3 mM ACC instead of ammonium sulphate as the N source and incubated at 30°C for 72 h (Wang et al., 2018). Samples for the assay were prepared according to the method described by the Belimov et al. (2005). Bacterial Acetyl-CoA Carboxylase (ACC) was quantified using the Bacterial ACCdase ELISA kit (Shanghai Huabang Biotechnology Co., Ltd.).

The 12 primer pairs (**Supplementary** **Table S3**) were employed to identify the antimicrobial genes of the microorganism in reference to the study of antibacterial synthases and regulatory genes by Li et al. (2021a). Fan et al. (2021) described the method, reaction conditions and procedure for genomic DNA extraction.

### 2.10 Optimization of culture conditions for fermentation of antagonistic bacteria

The impact of varying starting pH-buffers, temperature, incubation speed and inoculum volume on the growth (OD_600_) and the yield of inhibitory substances of *Bacillus siamensis* JSZ06 were investigated on the basis of the optimal growth medium using a one-way test. A one-way test was set up with six ndifferent pH values of 4.5, 5.5, 6.5, 7.5 and 8.5, and seven temperature levels of 21, 24, 27, 30, 33, 36 and 39 °C, six rotational speeds of 140, 160, 180, 200, 220 and 240 rpm, and five levels of inoculum concentrations of 1%, 2%, 3%, 4% and 5%. The inoculation was carried out in triplicates in 100 mL of medium for 72 h. Bacterial growth (OD_600_) and the inhibition of *Foc* TR4 growth by the fermentation broth were determined under different culture conditions to determine the optimal initial pH, temperature, rotation speed and inoculum concentration (Li et al., 2021c).

### 2.11 Determination of broad-spectrum antifungal activity of antagonist strain JSZ06 and its extracts

To assess the wide-ranging fungicidal effectiveness of this antagonistic strain and its extracts, we have chosen 10 species of plant pathogen fungus, among them *Botrytis cinerea* (ATCC 11542), *Rosellinia necatrix* (ATCC 28386), *Fusarium equiseti* (ATCC 26104), *Phytophthora nicotianae* (ATCC 66202), *Colletotrichum fragariae* (ATCC 58689), *Fusarium oxysporum*(ATCC MYA-1198), *Setosphaeria turcica* (ATCC 64836), *Fusarium oxysporum* f. sp. *lycopersici* (ATCC 16322), *Cercoseptoria zingiberis* (ATCC 46262) and *Fusarium solani* (ATCC 52628). All phytopathogenic bacteria were isolated and preserved by the Kunming Comprehensive Experimental Station of the National Green Fertilizer Industry Technology System. Four symmetrically positioned PDA plates were inoculated with antagonistic bacteria and strain fermentation extracts at a final concentration of 200 mg/L of extracts, followed by inoculation of 5 mm diameter discs of phytopathogenic fungi in the center of the PDA plates and incubated at 28°C for 7 days. The broad-spectrum resistance of JSZ06 strain and strain extracts was assessed by *in vitro* fungicidal action.

### 2.12 Influence of raw extract on *Foc* TR4 mycelium development

The growth inhibition of *Foc* TR4 was assessed by measuring the rate of mycelial growth. A variety of quantities of the 100% methanol-isolated strain extracts were then mixed with the purified PDA fluid, at the levels of 0.78, 1.56, 3.12, 6.25, 12.5, 25.0, 50.0, 100.0, and 200.0 mg/L. Each group’s negative control was the identical concentration of DMSO. In the middle of the plate, inoculate with 5 mm of *Foc* TR4 diameter patties. After 7 days at 28°C, the *Foc* TR4 growth diameter was assessed. Three copies of every treatment were used. A regression equation for toxicity was formulated using the method of least squares (Vanewijk and Hoekstra, 1993). Using the toxicity regression equation, the minimum concentration for 50% of the maximal effect (EC_50_) was calculated.

### 2.13 Effect of JSZ06 extract on the ultrastructure of *Foc* TR4 mycelia

The ultramorphological characterization of *Foc* TR4 mycelia, treated with extracts from the fermentation broth of strain JSZ06, was meticulously observed using Scanning Electron Microscopy (SEM; ZEISS Sigma 300, Germany). For the experimental setup, a 5 mm piece of *Foc* TR4 mycelium was inoculated at the centre of each PDA plate which contained 50 mg/mL of the strain extract. In contrast, DMSO with an equivalent concentration served as control. Following a 7-day incubation period at 28°C, a 1 cm^2^ block of agar, inclusive of the mycelium, was carefully excised from the periphery of the *Foc* TR4 growth. The ultramorphology of both the extract-treated *Foc* TR4 mycelium and the control mycelium was subsequently analysed via SEM (Fan et al., 2021).

### 2.14 Impact of strain exposure on the mycelial cell structure of *Foc* TR4

The effect of extracts on the cell architecture in *Foc* TR4 was observed using a TEM (HT7700, Hitachi Ltd, Japan). Referring to the relevant methodology (Li et al., 2021c), samples were collected, fixed, and dehydrated. After 12 h at 35°C, 24 h at 45°C, and 48 h at 60°C, the samples were aggregated after being implanted in Epon812 resin. The material was then sliced into 80 nm sheets. Uranyl acetate and lead citrate solution were used to double-stain these sections. Mycelial cells’ ultrastructure was examined by TEM after the samples had dried naturally at room temperature.

### 2.15 Impact of strain extracts on *Foc* TR 4 spore germination

We determined the effect of *Bacillus siamensis* JSZ06 extracts on the germination rate of *Foc* TR4 spores. Extracts of strains with concentrations of 1, 2, 4, and 8 × EC_50_, were thoroughly mixed with 45 µL of *Foc* TR4 spore suspension (1.0 × 10^6^ CFU/mL) on concave slides and then allowed to stand for 12 h at 28°C. Subsequently, the extracts were mixed with 45 µL of *Foc* TR4 spore suspension (1.0 × 10^6^ CFU/mL). Equivalent concentrations of dimethyl sulfoxide-treated spore suspensions were used as control. All experiments were repeated three times. The germination of 100 spores on each concave slide was examined using a light inverted microscope. Inhibition was assessed on the basis of spore germination (Zou et al., 2021).

### 2.16 Annotation of functional genes

The identification of protein encoding sequences (CDS) within the genomic structure was achieved using Glimmer v3.02 analysis tools (Delcher et al., 2007). Based on the whole genome sequence, estimation of gain was made of the G + C content of the strain genome. The bacterial genomes were annotated using GO (cloud.majorbio.com), COG (http://www.ncbi.nlm.nih.gov/COG) and KEGG (http://www.genome.jp/kegg/) databases (Li et al., 2021c). Employing the web-based tool antiSMASH, the prediction of secondary metabolite gene clusters in genomes was constructed (Huang et al., 2019).

### 2.17 Metabolite composition analysis of JSZ06

Metabolites of anti-*Foc* RT4 in JSZ06 fermentation broth were determined using a metabolomic approach (Yun et al., 2023). The analysis of the specimens was performed with an Ultra High Pressure Liquid Chromatography (Agilent 1290 Infinity, USA) tandem Fourier Transform Mass Spectrometer (AB Triple TOF 6600, USA) UHPLC -Q Exactive system. Sample extraction and detection was carried out using LC-MS/MS (Wang et al., 2019; Xie et al., 2019; Yun et al., 2023). Metabolite profiles were generated by aligning mass spectrometry data with data from publicly available metabolic databases, namely HMDB (http://www.hmdb.ca/), Metlin (https://metlin.scripps.edu/), and Lipidmaps (http://www.lipidmaps.org). Metabolic routes, via KEGG database pathway annotation, were also performed to identify the routes implicated in the compounds. The data analysis was constructed using the Majorbio Choud platform, which was available for free online (cloud.majorbio.com) (Ren et al., 2022).

### 2.18 Banana seedling experiment with pot inoculation

From August to October 2023, the effectiveness of the fermentation solution of the antagonistic strain against banana wilt was assessed at 70% relative humidity and a standard room temperature of 28°C in a controlled environment. He et al. (2021) contributed the overexpression procedure of the fluorescent green protein gene *Foc* TR4 (GFP-*Foc* TR4). One seedling of histocultured ‘Baxi’ banana (AAA) from the seedling bag was transplanted into a plastic pot (5 cm × 8 cm) containing 2 kg of sterile soil (Fan et al., 2021). Four different types of intervention were designed as follows: distilled water, GFP-*Foc* TR4 (1.0 × 10^6^ CFU/ml), antagonist strain (1.0 × 10^8^ CFU/ml) + GFP-*Foc* TR4 (1.0 × 10^6^ CFU/ml), and antagonist strain (1.0 × 10^8^ CFU/ml). As recommended (Fan et al., 2021), 100 mL of the combination were applied to the roots of banana plants. For a total of 45 days, the development of banana seedlings was measured every 7 days in 30 plants per treatment.

### 2.19 Evaluation of biological control and growth promotion effects

On days 0, 7, 14, 21 and 45 of inoculation, banana roots and bulbs were collected and cut as finely as possible. A confocal laser scanning microscopy (Leica TCS-SP, Wetzlar, Germany) was used to rapidly detect the level of GFP-*Foc* TR4 infection and settlement (Zhou et al., 2023).

Forty-five days after inoculation, the infection of banana leaves and bulbs was observed and recorded separately. The disease index and control effect of each group were computed following the methods of Fan et al. (2021) and Zhou et al. (2023). The following standards for assessing leaf diseases were applied: grade 0: no indications; grade 1: no more than 50% of the whole area with wilting of true leaves and cotyledons; grade 2: more than 50% of the whole area with wilting of true leaves and cotyledons; grade 3: just the growth tips remained, with the leaves withered or dead; grade 4: the whole plant seemed very withered or dead. Bulb disease grading: grade 0: no lesions on the bulb; grade 1: the area of lesions on the bulb is 1% - 10%; grade 2: the bulb lesion area is 11%~30%; grade 3: the bulb lesion area is 31%~50%; grade 4: the bulb lesion area is more than 50%. Plant height, fresh weight, stem thickness, leaf length and leaf width of the banana plants in all treatments were measured and recorded on days 7, 21 and 45 after inoculation.

### 2.20 Data analysis

Data were subjected to one-way ANOVA, with the significance of differences between treatments being assessed using Duncan’s multiple range test (*P*< 0.05). Therefore, both Excel and SPSS Statistics 20.0 software were utilized for statistical analysis and orthogonal experimental design. Consequently, graphical representations were created using Origin 2021 and GraphPad Prism 8 software.

## 3 Results

### 3.1 Isolation of endophytes

**Figure 1:**
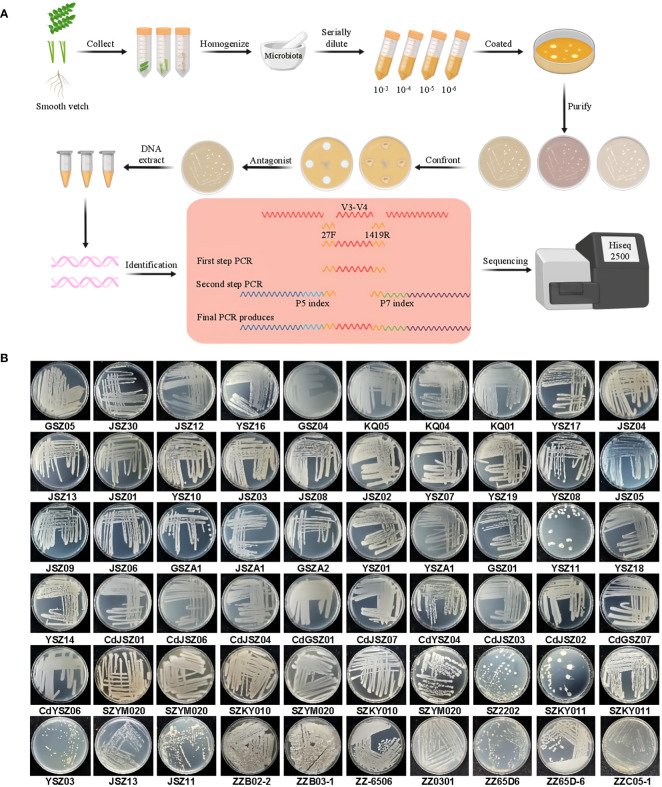
Growth characteristics of 60 endophytic bacteria isolated from smooth vetch. **(A)** Smooth vetch can be cultivated for the isolation and identification of endophytic bacteria for detailed analysis of experimental procedures. **(B)** Growth characteristics of 60 strains of endophytic bacteria.

Smooth vetch antagonistic endophytes were isolated using a correlation procedure at the green manure long-term localization test site in Songming County, Kunming, Yunnan Province (102° 41’E, 25° 28’N) (**Figure 1A**). Sixty strains of endophytic bacteria were isolated using taproots, branches and leaves of smooth vetch (**Supplementary Figure S1**). All strains were conserved at the Kunming Comprehensive Experimental Station, National Green Fertilizer Industry Research and Development Center, Kunming, China (**Figure 1B**).

### 3.2 Determination of antifungal activity of isolated endophytes

Firstly, all isolated endophytic strains were verified against *Foc* TR4. The inhibitory capacity of mycelial growth was determined for TR4. Eleven of the 60 endophytic strains exhibited a significant antifungal efficacy in the plate antagonism test (**Supplementary Figure S2A**), representing 18.30% of screened endophytes. Furthermore, the suppressive effects of the 11 antagonistic endophytic strains against *Foc* TR4 were assessed. The strain numbered JSZ06 showed the strongest inhibitory activity with 70.20% inhibition.

Simple fermentation of 11 endophytic strains from a preliminary screening with significant antagonistic effects was carried out. Their crude extracts were further investigated for their antagonistic ability against Foc TR4. The results showed that the crude extracts of all strains inhibited *Foc* TR4 in the range of 35.36-70.60% (**Supplementary Figure S2B**). Strong inhibitory circle size was observed in the extracts of five isolates (JSZ06, JSZ01, JSZ02, YSZ10 and YSZ19). The inhibition rates were 70.56, 58.69, 57.14, 60.00 and 56.43%, respectively. Combining the results of initial and rescreening, strain JSZ06 was selected as the most suitable for subsequent studies.

### 3.3 Characteristics and identification of strain JSZ06

#### 3.3.1 Characteristics of culture

The growth characteristics of strain JSZ06 have been measured on 7 selected media (**Supplementary** **Table S1**). It grew well on LB and TSA media and moderately on the rest of the media except R2A, CDA and Gause’s no.1 media. The colonies showed an overall colour ranging from yellow to grey on all the media. Colonies were light yellow on PDA and TSA media, light grey on LB, yellowish white on NA, beige on let R2A and lemon yellow on CDA and Gause’s no.1. Both irritating odour and biofilm were produced on the LB medium, and the colony bulge was the largest in diameter. Multiple notched edges, irregular growth, rough appearance, opacity, and wrinkled surface of the colonies were observed on all media, this provides an additional means of distinguishing the genus *Bacillus* (He et al., 2021).

#### 3.3.2 Features of physiological and chemical systems

In order to further investigate the physiological and biochemical activities of JSZ06, we assayed JSZ06 using the Biolog method. The results showed that in terms of physicochemical aspects, the strain was able to be positive at pH 5 and pH 6, and at 1% NaCl, 4% NaCl, and 8% NaCl, i.e., JSZ06 could grow normally within these limits. In terms of sugar utilization, JSZ06 was able to catabolize and utilize D-Maltose, D-Trehalose, D-Cellobiose, α-D-Glucose, D-Mannose, D-Fructose and other sugars, on the other hand it could not fully utilize D-Galactose, D-Turanose, Stachyose and others. In addition, JSZ06 can also utilized D-sorbitol, D-mannitol and glycerol, but not D-arabinitol. In terms of amino acid utilization, a variety of amino acids including L-alanine, L-arginine, L-aspartic acid, and L-glutamic acid can be utilized, but not L-histidine, L-pyroglutamic acid, and D-serine (**Supplementary** **Table S2**). In summary, JSZ06 can adapt to a variety of micro-environments and can decompose and utilize different kinds of organic matter, which is consistent with the physiological and biochemical characteristics of *Bacillus* spp.

#### 3.3.3 Genomic characterization and phylogenetic analysis of isolates

**Figure 2:**
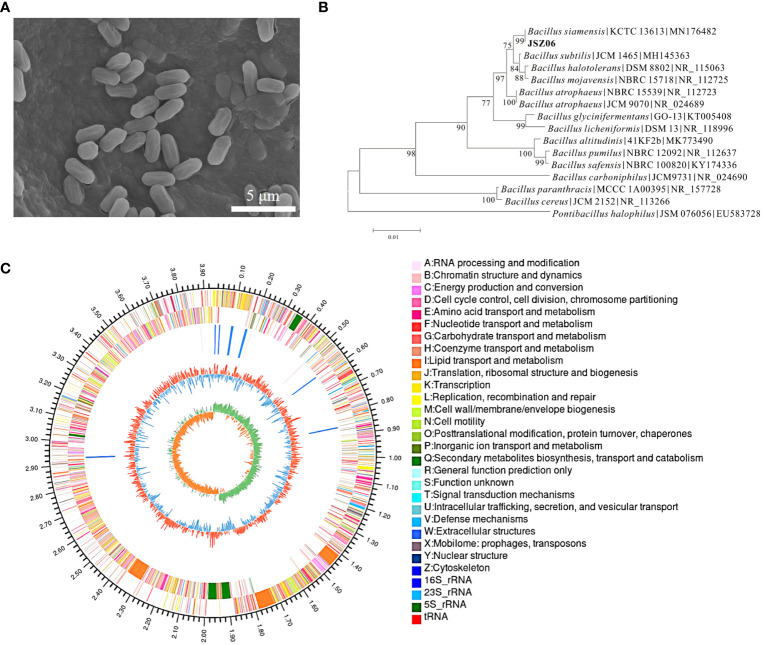
Characterization of strain JSZ06. **(A)** Scanning electron micrograph of JSZ06. **(B)** Phylogenetic tree of 16S rRNA gene of JSZ06. **(C)** JSZ06 strain genome ring diagram. Genome size identification is represented by the outermost circle of the ring diagram; CDS on the positive and negative strands are represented by the second and third circles; rRNA and tRNA are represented by the fourth circle; GC content is represented by the fifth circle; and the GC-Skew value is represented by the innermost circle.

Electron microscope scanning (SEM) showed that strain JSZ06 organisms were short rod-shaped or kidney-shaped, with an unsmooth bacterial surface. Their lengths ranged from 0.75 to 1.20 µm and widths from 0.52 to 0.75 µm (**Figure 2A**). We then analysed the 16S rRNA region of the whole genome of JSZ06 with the aim of precisely identifying the species (**Figure 1A**). The strain JSZ06’s 16 S RNA sequences compared to the GenBank library revealed the greatest degree of similarity to *Bacillus siamensis*. Predicted on the sequences’ evolutionary tree, JSZ06 was found to be similar to *Bacillus siamensis* (GenBank accession number: MN 176482). By examining the morphological and genetic features mentioned above, bacterium JSZ06 was recognized as *Bacillus siamensis* (GenBank accession number: PP226927) (**Figure 2B**). The whole genome of the antagonist bacterium JSZ06 was sequenced with a total distance of 3,935,105 bp and a 46.5% G + C ratio (Login number: CP143783). The 3,749 genes that code for proteins, 86 tRNA genes, and 27 rRNA genes made up the whole genome (**Figure 2C**).

### 3.4 Detection of antibiotic synthesis gene of strain JSZ06 and its functional test results

**Figure 3:**
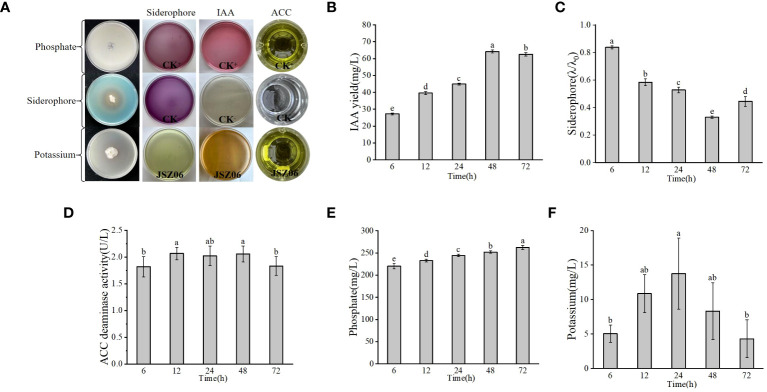
Evaluation of PGP traits and genetic detection of antimicrobial active substances in plant endophytes. **(A)** PGP traits of JSZ06. **(B–F)** Determination of IAA, iron carrier, ACC, phosphate solubilisation and mineral potassium solubilisation yields during culture of endophyte JSZ06. The error bars represent the standard deviations of the means from three independent experiments. Different lowercase letters indicate a significant difference at the level of *P*< 0.05. Production of siderophores (λ/λ_0_) ranges from small (0.8-1.0), low (0.6-0.8), moderate (0.4-0.6), high (0.2-0.4), to extremely high (0-0.2).

The PGP traits of strain JSZ06 were assessed and represented in **Figure 3A**. Strain JSZ06 was able to produce IAA at 6 h of incubation and up to 64.23 mg/L at 48 h with a total IAA production ranging from 27.27 to 64.23 mg/L (**Figure 3B**). A CAS assay is shown in **Figure 3C** and the λ/λ0 value of JSZ06 after 48 h was 0.33, which is in the category of a high iron carrier producing bacteria. Also, ACC utilisation attained a peak of 2.07 U/L at 48 h (**Figure 3D**). In addition, strain JSZ06 was able to solubilise mineral phosphate and mineral potassium up to a maximum of 262.47 mg/L and 13.77 mg/L (**Figures 3E, F**), which indicated that the endophytic strain JSZ06 possesses good growth-promoting properties and has the capacity to enhance stress tolerance in plants and encourage plant development.

PCR of the strain showed that strain JSZ06 had seven Non-Ribosomal Peptide Synthetases (NRPS) producing genes (**Supplementary Figure S3**; **Supplementary Table S4**), four Polyketide Synthase genes (PKS) for four Polyketide metabolites, and growth-promoting related genes for Ribosomal Peptide Synthetases (RPS), including *srfAA*, *yndJ*, *fenD*, *ituC*, *yngG*, *bamD*, *dhb* and *dfn*, *bae*, *mln*, *bac* and *sboA*. This is instructive for subsequent genome-wide prediction and isolation and identification of antimicrobial substances.

### 3.5 Inhibitory growth optimisation of the antagonist bacterium JSZ06

The process of creating bactericidal materials by the antagonist JSZ06 was favoured when the culture conditions were at a suitable pH level, fermentation temperature, culture speed and inoculum amount. At a pH below 4.5 and above 8.5, the concentration in the broth was considerably less than in the other concentration gradients, and the OD_600_ values were only 2.06 and 2.10. The result suggested that both too low and too high pH would inhibit the growth of the strain. At the pH of 7.5, the OD_600_ value of the fermentation broth was the highest at 2.58 and was the highest inhibition rate of fermentation broth against pathogens at 61.46% (**Supplementary Figure S4A**). When the temperature was at 33°C, The strain’s highest OD_600_ value for broth production was 2.64, and the inhibition rate was 64.48%, which was the best effect (**Supplementary Figure S4B**). Different rotational speeds had different effects on the OD_600_ value and the inhibition rate of the incubation fluid of the microorganisms. The OD_600_ values of the fermentation broths were similar when the rotational speeds were 180 and 200 rpm, but the inhibition rate was larger at 200 rpm, which was 62.66% (**Supplementary Figure S4C**). Whereas, when the inoculum was 2%, the strain fermentation broth OD600 value and inhibition rate were higher at 2.73 and 56.58%, respectively (**Supplementary Figure S4D**). Therefore, the optimal pH, fermentation temperature, culture speed and inoculum amount of the strain were comprehensively defined as 7.5, 33°C, 200 rpm and 2.0%, respectively.

### 3.6 Assay for wide-spectrum fungicide action

**Figure 4:**
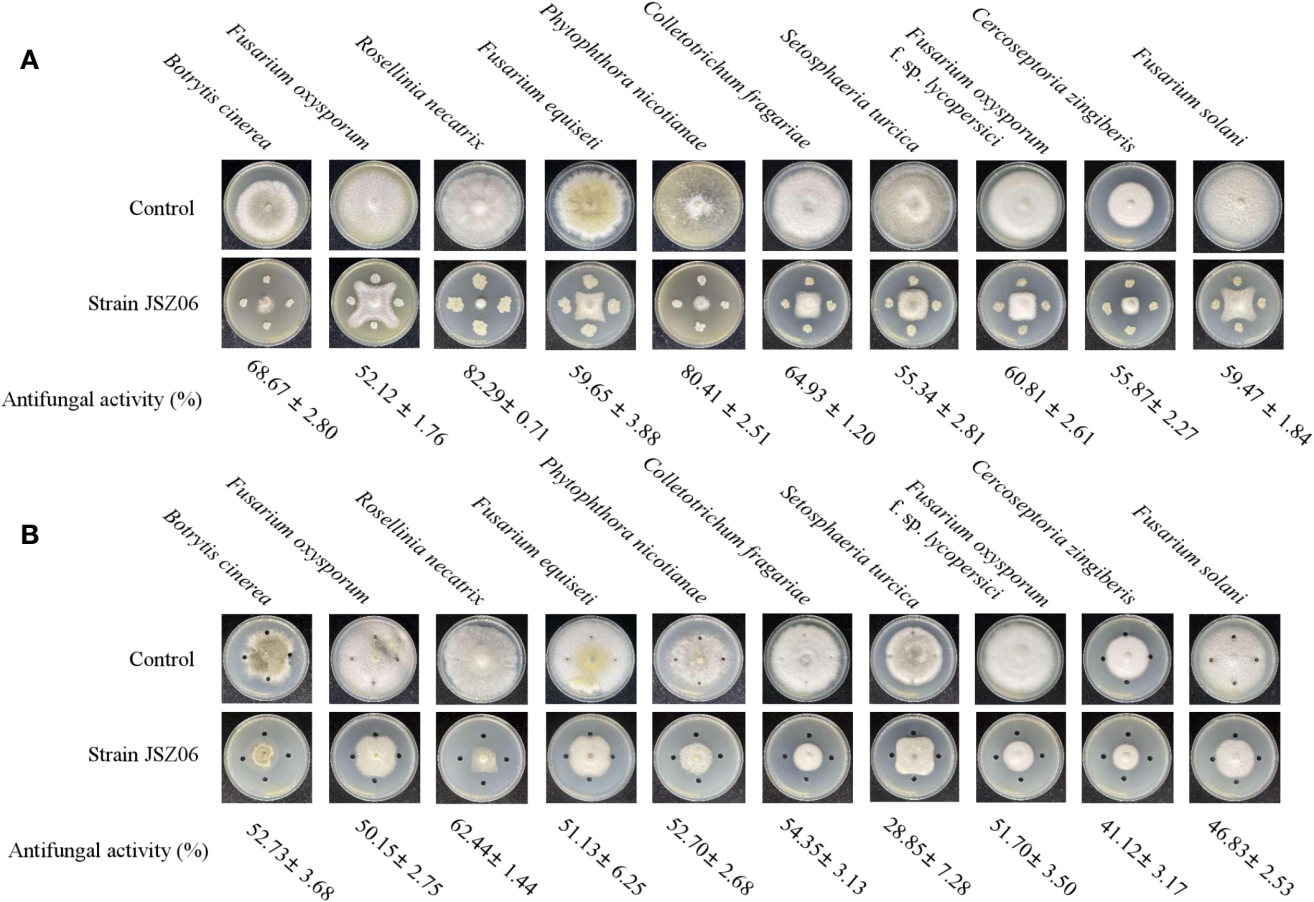
Wide-ranging antifungal action of JSZ06, an antagonistic fungus. **(A)** The ability of strain JSZ06 to inhibit a certain plant-pathogenic fungus. **(B)** Crude extract’s antifungal efficacy against a specific plant-pathogenic fungus.

Strain JSZ06 and its extracts showed good broad-spectrum resistance and significantly inhibited the growth of 10 phytopathogenic bacteria (**Figure 4**). Strain JSZ06 showed the highest inhibition of 82.29% against *Rosellinia necatrix* and the lowest inhibition of 52.12 against *Fusarium oxysporum* (**Figure 4A**). The extract of strain JSZ06 showed the highest inhibition of 62.44% against *Rosellinia necatrix* and the lowest inhibition of 28.85% against *Setosphaeria turcica* (**Figure 4B**).

### 3.7 Inhibition of mycelial growth of *Foc* TR4 by extracts of strain JSZ06

**Figure 5:**
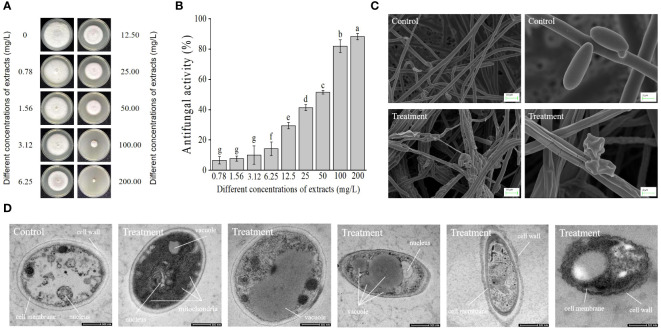
Impact of strain JSZ06 extracts on *Foc* TR4 mycelial development and ultramorphology. **(A)** *Foc* TR4 growth inhibition on PDA medium after different extraction dosages were applied. **(B)** Growth inhibition rate of *Foc* TR4 after treatment with different extraction doses. **(C)** SEM observation of the mycelial morphology of *Foc* TR4. **(D)** TEM micrographs showing 50 mg/L of strain JSZ06 treated *Foc* TR4 cells. The *Foc* TR4 mycelium treated with 10% DMSO is characterized by blanks. *Foc* TR4 treated with 50 mg/L of strain JSZ06 extract and its mycelial morphology. At the significance threshold of *P*< 0.05, different lowercase letters indicate a significant difference.

Seven days later, extracts from strain JSZ06 greatly slowed the development of *Foc* TR4’s mycelium. The inhibitory ability became more pronounced with a gradual increase in the concentration of the extract (**Figure 5A**). In contrast to the *Foc* TR4 development width in the control plate (7.00 cm ± 0.20), the mycelial growth diameter in the 12.5 mg/L extract treatment group was significantly reduced to 4.94 cm ± 0.07. Upon achieving 200 mg/L of strain JSZ06 extract level, considerable suppression of *Foc* TR4 development was observed, with a suppression rate of 88.3% ± 0.02 (**Figure 5B**). The toxicity regression equation was further established (*y* = 1.17861*x* + 3.27375, *R* ^2^ = 0.94537), and the JSZ06 extract’s EC_50_ value against *Foc* TR4 was 29.15 mg/L, was defined as 1 × EC_50_ in the follow-up study.

### 3.8 Effects of JSZ06 extracts on *Foc* TR4’s mycelial morphological features

By electron microscopy scanning, the *Foc* TR4 mycelium sampled from the inhibitory zone’s edge were different to the control. Normal mycelia in the control group had a smooth surface and normal, smooth, and plump tops. The mycelia subjected to JSZ06 extract treatment, were hard, swollen irregularly, and the cell walls had crumbled. The branches on the mycelial tips were in the form of webbed feet and had spherical, fusiform swelling (**Figure 5C**). Furthermore, the exposed surface of the *Foc* TR4 spores to the strain extracts exhibited depression and deformation. The spore surface in the control group, however, was uniform, regular, and normal, indicating that the extract significantly impacted *Foc* TR4 growth (**Figure 5C**).

### 3.9 Influence of JSZ06 strain isolates on the ultrastructure of *Foc* TR4

The texture of the *Foc* TR4 mycelium after 50 mg/L of treatment of the extract was visualized by transmission electron microscopy. The untreated treatment’s cell walls and membranes were still visible. There were also several undamaged organelles on display, including the nucleus and vacuole (**Figure 5D**). After treatment with extracts of strain JSZ06, the number of mitochondria with aberrant cell walls increased significantly and the nucleus disintegrated (**Figure 5D**). The vacuole grew until it eventually ruptured (**Figure 5D**). Organizational disarray, matrix blurring, and organelle lysis were observed in *Foc* TR4 cells, with plasma-wall segregation, and disruption and progressive deterioration of the cell membrane and membrane integrity (**Figure 5D**).

### 3.10 Impact of strain extracts on *Foc* TR4 spore morphological features and germination

In this study, the impact of the crude extract from the antagonistic strain JSZ06 on both the growth and spore formation of the pathogenic fungus *Foc* TR4 was thoroughly investigated (**Supplementary Figure S5A**). A considerable variation in the impact of various crude extract levels on the development of spores (*P*< 0.05) was found. As the concentration of the bacterial strain extract increases, the growth rate of spores reduces as a consequence. The highest concentration that proved most effective for the growth of spores was 8 × EC_50_. This fungus’ maximal spore development inhibition towards *Foc* TR4 was 10%. As a result, the 10% DMSO (v/v) treatment served as a means of control and had no effect on the harmful fungi’s ability to germinate.

The shape of *Foc* TR4 before and after 1 ×, 2 ×, 4 × and 8 × EC_50_ treatment was further studied by SEM (**Supplementary Figure S5B**). The untreated spores that received a 10% DMSO treatment had a smooth surface and were undamaged. Greater morphological changes, such as stretching, loss of volume, disintegration, folding, bending, and cell rupture, were observed in spores treated with 2 ×, 4 ×, and 8 × EC_50_ crude extracts, significantly disrupting the normal morphology and integrity of the spores.

### 3.11 Genome analysis using bioinformatics

By annotation, GO, COG, and KEGG groups were assigned to 2,286; 3,077, and 2,377 encoding genetics, correspondingly. In the GO category, the following types of anticipated genes were identified: molecular roles (1,861), parts of cells (1,145), and processes in biology (1,235) (**Supplementary Figure S6A**). These genes were divided into four groups of 23 genes under the COG category. The biggest subcategory was physiology (1,479), which was followed by communication and processes in cells (957) and information storage and processing (641). Ultimately, 392 genes were classified as inadequately described (**Supplementary Figure S6B**). Based on KEGG evaluation, 41 pathways including 2,377 genes that encode proteins were identified (**Supplementary Figure S6C**). The major components of resistance to disease include the processing of signals, secondary metabolite production, and terpenoids and polyketide degradation. The genome of bacterium JSZ06 was projected to include twelve metabolic gene groups by antiSMASH software compared with GenBank, including NRPS gene cluster, PKS-like gene cluster, gene cluster for terpenes, gene cluster lanthipeptide-class-ii, TransAT-PKS gene cluster, the T3PKS gene cluster, Betalactone gene cluster, NRP-metallophore gene cluster and RIPP-like gene cluster (**Supplementary Table S5**). Eight of these gene clusters could be compared to similar gene clusters capable of producing Bacillaene, Fengycin, Difficidin, Bacillibactin, Surfactin, Butirosin A, B, Macrolactin H, bacilysin and other anti-substances (**Supplementary Figure S8**). The corresponding chemical structural formulae are shown in **Supplementary Figure S7**.

### 3.12 Detection of bioactive compounds by LC-MS/MS from strain JSZ06 extract

JSZ06 metabolites’ anti-*Foc* TR4 active components were examined using LC-MS. The positive and negative ion flow chromatograms are shown in **Supplementary Figure S9**. The final statistical results showed that, via missing value filtering of the initial data, lacking appreciate converting, data normalization, Quality Control (QC) confirmation, and information change, the POS (+) and NEG (-) models produced 1,259 and 1,050 metabolites, accordingly, of which 1,086 and 1,021 compounds have been added to publicly accessible databases like HMDB and Metlin (**Table 1**).

**Table 1:** Data about detected metabolites.

Ion Mode	Every Peak	Determined Metabolites	Libraries’ Metabolites	KEGG’s metabolites
POS	6751	1259	1080	542
NEG	10919	1050	1016	449
Total	17670	2309	2096	991

Number of POS and NEG; number of metabolites annotated in the corresponding database.

Assigning the above metabolites to the KEGG and HMDB databases, 542 and 449 metabolites were categorized into 18 KEGG secondary pathways, respectively, of which 509 metabolites were categorized as “metabolites”. Among the metabolites, the most abundant was “Amino acid metabolis”, followed by “Biosynthesis of other secondary metabolites”, “Metabolism of cofactors and vitamins”, “Xenobiotics biodegradation and metabolism”, “Lipid metabolism”, “Metabolism of other amino acids” and “Carbohydrate metabolism” processes (**Supplementary Figure S10A**). Within the KEGG secondary pathway, 63 metabolites are designated as “biosynthesis of other secondary metabolites”. Of these, there are a variety of antimicrobial metabolites in the KEGG Significant Pathway Statistics modeling synthesis pathway, including “biosynthesis of various other secondary metabolites” (13), “biosynthesis of various alkaloids” (12), “biosynthesis of various antibiotics” (10), “biosynthesis of neomycin, kanamycin, and gentamicin” (6), “biosynthesis of carbapenems” (4), “Biosynthesis of Penicillins and Cephalosporins” (4), “Vancomycin Resistance” (3), “Staurosporine Biosynthesis” (3), “Novobiocin Biosynthesis” (3), “Taurine and Low Taurine Metabolism” (3), “Prodigiosin Biosynthesis” (3), and others (**Supplementary Figure S11**). These pathways involved in the synthesis of antimicrobial metabolites play crucial roles in exerting antagonism against pathogenic bacteria.

The 1080 and 1016 metabolites identified in the secondary analysis were cross-referenced with the 16 HMDB superclasses available in the HMDB database. Among these, 657 (31.35%), 443 (21.14%), and 375 (17.89%) metabolites were categorized under “Organic acids and derivatives,” “Lipids and lipid-like molecules,” and “Organoheterocyclic compounds,” respectively. These classifications constituted the predominant portion within all categories, as illustrated in (**Supplementary Figure S10B**).

### 3.13 Identification of antimicrobial metabolites of strains

To further identify antimicrobial substances in the fermentation broth, a total of 2,309 identified substances were analyzed. The findings revealed a wide range of main intermediates in the strain JSZ06 extract. Several antibacterial compounds were found among the secondary metabolites, all of which have demonstrated antimicrobial activity. These substances include Validamycin A, Neamine, Apramycin, Hygromycin B, and Surfactin in the POS, and Cephamycin C, Neomycin in the NEG, as well as Novobiocin, Oxytetracycline, Ribostamycin, Aerobactin, and others (**Supplementary Table S6**). These results suggest that strain JSZ06 is an advantageous strain with significant potential for biocontrol, as it produces a variety of antagonistic substances capable of preventing the spread of harmful fungi.

### 3.14 Effect of fermentation broth of strain JSZ06 on the biological prevention of banana wilt disease

**Figure 6:**
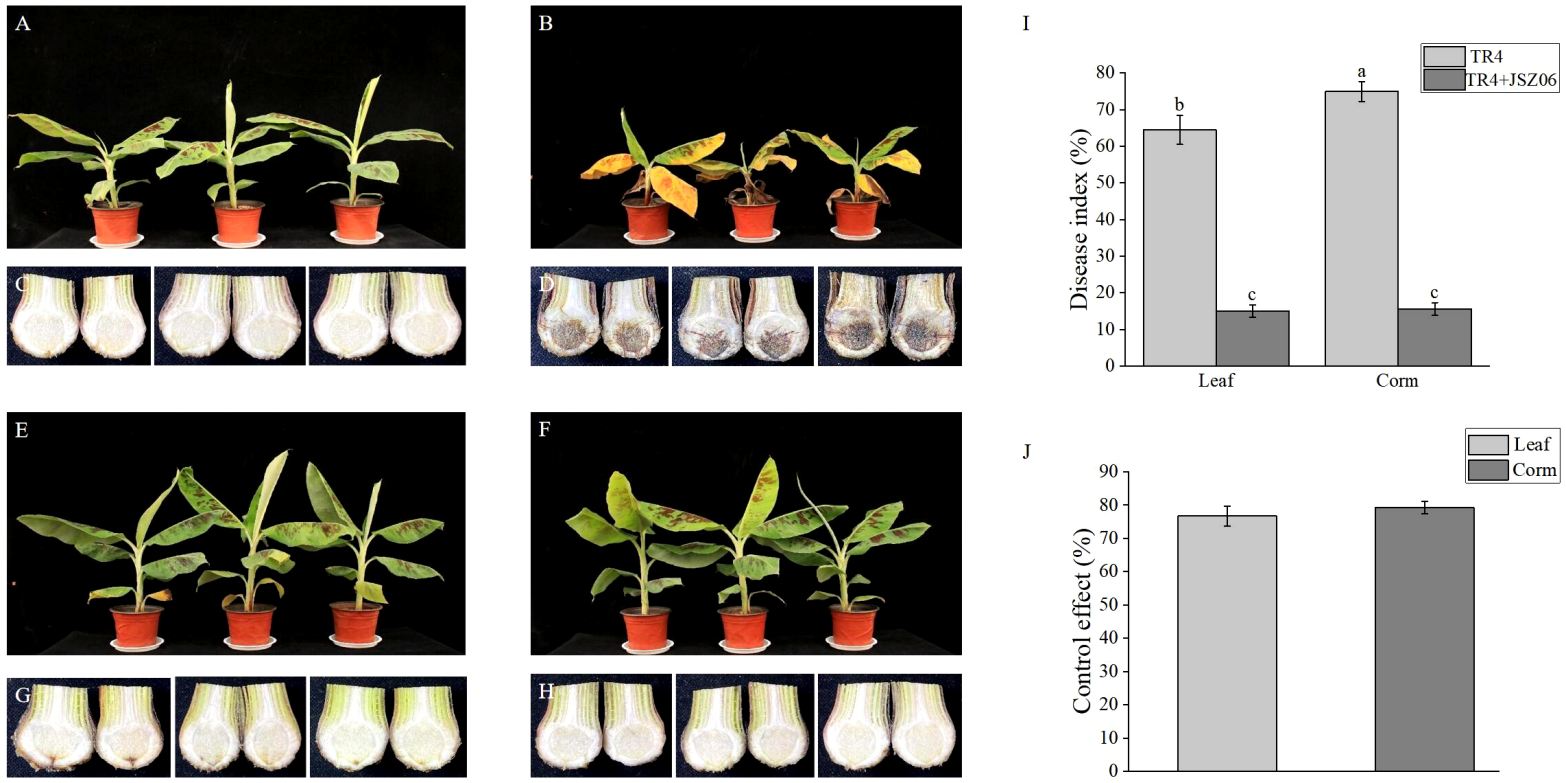
The growth and resistance to *Foc* TR4 in banana seedlings were affected by the antagonist strain JSZ06 fermentation broth. **(A, C)** Blank control without the JSZ06 or TR4 vaccination. The injection of TR4 (1 × 10^6^ cfu/mL) in **(B, D)**. Inoculation with JSZ06 (1 × 10^8^ cfu/mL) and TR4 (1 × 10^6^ cfu/mL) **(E, G)**. JSZ06 (1 × 10^8^ cfu/mL) inoculation **(F, H)**. **(I)** Indexes of banana leaf and bulb disease 45 days after *Foc* TR4 inoculation. **(J)** JSZ06 inoculation’s controlling impact on banana bulbs and leaves. At the significance threshold of *P*< 0.05, different lowercase letters indicate a significant difference.

After a total of 45 days of inoculation, severe wilting and necrosis symptoms were observed on leaves of the banana plants treated solely with *Foc* TR4 (**Figure 6B**). In contrast, banana leaves treated simultaneously with the fermentation broth of strain JSZ06 and *Foc* TR4 remained green and healthy (**Figure 6E**). Banana leaves treated with sterile water and the fermentation broth of strain JSZ06 separately showed no signs of disease (**Figures 6A, F**). Upon longitudinal sectioning of the banana pseudostems subjected to different treatments, those treated with *Foc* TR4 exhibited wilting, with the longitudinal sections turning brown-black (**Figure 6D**), while no significant infection was evident in the banana pseudostems treated with the fermentation broth of strain JSZ06 along with *Foc* TR4 (**Figure 6G**). Additionally, there were no signs of infection on pseudostems treated solely with sterile water or strain JSZ06 (**Figures 6C, H**). Furthermore, following treatment with the fermentation broth of strain JSZ06, both the disease severity indices of banana leaves and pseudostems showed a significant decrease (**Figure 6I**), with biocontrol efficiencies reaching 76.71% and 79.25%, respectively (**Figure 6J**).

### 3.15 Effect of the antagonistic bacterium JSZ06 on TR4 infestation of banana roots and bulbs

**Figure 7:**
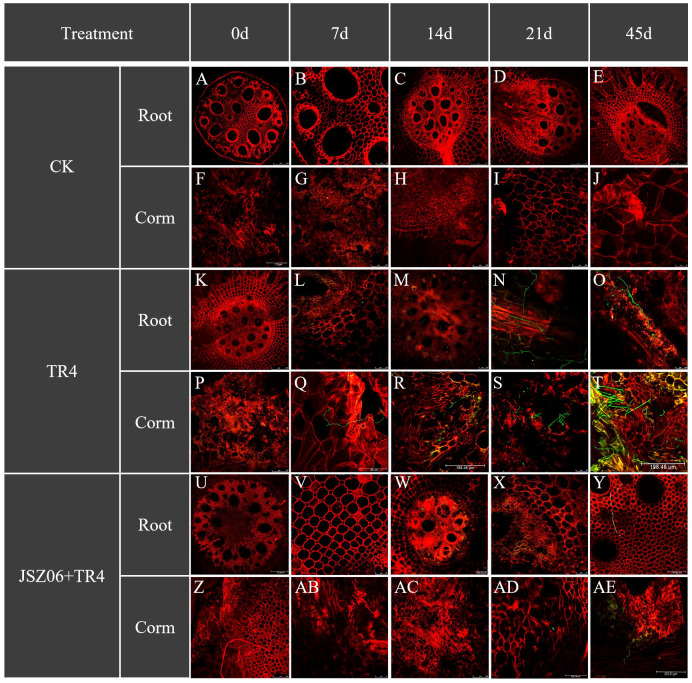
Infestation process of banana roots and bulbs at different time points (0 dpi, 7 dpi, 14 dpi, 21 dpi and 45 dpi) after inoculation with GFP-*Foc* TR4 and strain JSZ06. Bar = 100 μm in **(N, O)**; Bar = 126.14 μm in **(F, AD)**; Bar = 198.48 μm in **(R, T)**; Bar = 250 μm in **(A–E, G–M, P, S, U–Z, AB, AC)**; Bar = 253.31 μm in **(Q, AE)**.

Forty-five days after inoculation with GFP-*Foc* TR4, JSZ06 drastically reduced the incidence of *Foc* TR4 infection on bananas (**Figure 7**). TR4 presence was detected in both banana roots and bulbs 7 days post-inoculation with TR4 alone. Conversely, in the JSZ06 treatment group, no TR4 hyphae were observed. Following a 14-day inoculation period, a significant amount of mycelia was observed in the banana roots and bulbs that had received TR4 injections only. However, mycelial growth was limited in banana roots treated with JSZ06 inoculation. Subsequent observations at 21 and 45 days post-inoculation revealed progressively larger mycelial infestations in the roots and bulbs of bananas treated with TR4 alone. In contrast, minimal mycelium was observed in both roots and bulbs of bananas inoculated with JSZ06, with the highest infestation observed only at 45 days, but still considerably lower compared to the TR4-alone treatment. Based on the tracking of TR4 infestation over time, we hypothesized that treatment with strain JSZ06 could gradually enhance banana plant resistance to TR4 mycelial invasion.

### 3.16 Effect of fermentation broth of strain JSZ06 on the development of banana plants

**Figure 8:**
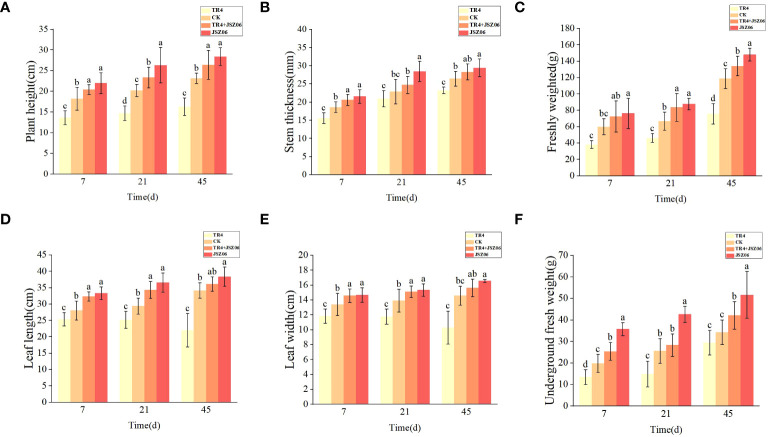
The development and growth of banana seedlings was greatly enhanced by the antagonist bacteria JSZ06 fermentation broth. Physiological indicators in the control and treatments of banana seedlings: **(A)** plant height, **(B)** stem thickness, **(C)** above-ground biomass, **(D)** leaf length, **(E)** leaf width, and **(F)** root biomass, measured after 7, 21, and 45 days, respectively. At the significance threshold of P< 0.05, different lowercase letters indicate a significant difference.

Pot trials were conducted to assess the impact of JSZ06 broth fermentation on banana development. Application of JSZ06 fermentation broth treatment resulted in a significant increase in plant height, leaf size, stem thickness, leaf width, above-ground biomass, and below-ground biomass during the banana growth period compared to both the control and TR4-alone treatments (**Figures 8A–F**). It is noteworthy that the application of strain JSZ06 in the JSZ06 + TR4 treatment still significantly enhanced banana growth compared to the TR4-alone treatment, albeit to a lesser extent than the JSZ06-alone treatment. Thus, the antagonist bacterium JSZ06 was able to mitigate the inhibitory effect of TR4 on banana growth and promote growth to a certain extent.

### 3.17 Conceptual modeling

**Figure 9:**
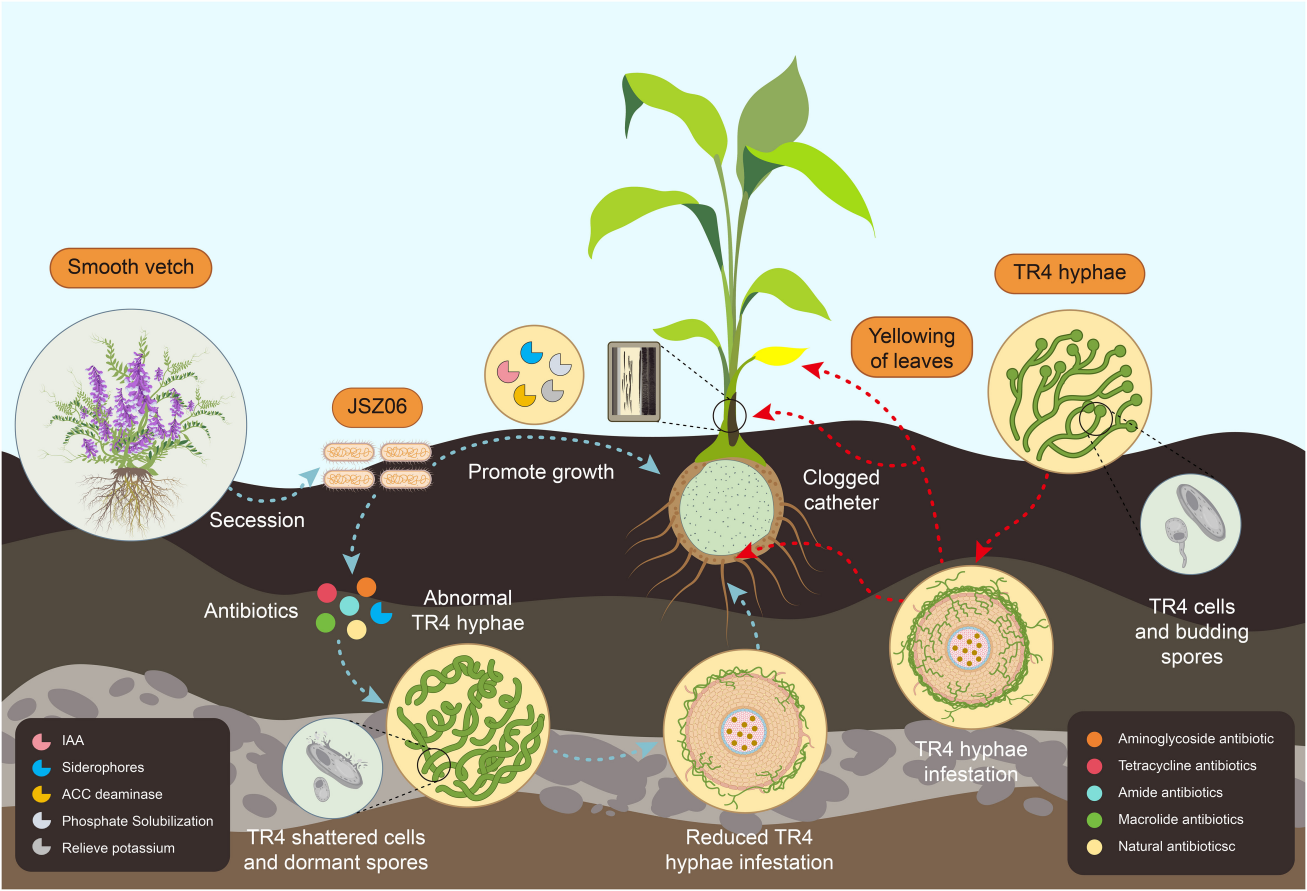
A conceptual model of the mechanism of action of strain JSZ06 in reducing banana wilt disease.

Based on the aforementioned results, a conceptual model illustrating the mechanism of action of the biocontrol bacterium JSZ06 against banana wilt was developed (**Figure 9**). The conceptual model indicates that JSZ06 reduces the incidence of banana wilt by producing antibiotic-type bacteriostatic agents that disrupt the structure of *Foc* TR4 mycelia and inhibit spore germination, thereby reducing the infestation of TR4 mycelia on bananas. Additionally, JSZ06 promotes banana growth by producing beneficial substances that enhance the plant’s resistance to pathogenic bacteria. This symbiotic relationship contributes to the overall health and vigor of the banana plant, aiding in its defense against wilt disease.

## 4 Discussion

*Fusarium oxysporum* f. sp. *cubense* Tropical Race 4 (*Foc* TR4), a soil-borne fungus, poses significant threats to banana crops and currently lacks practical control methods (Dita et al., 2010). Sources of infection for this pathogen include bacterial sucking buds, diseased plant residues, and contaminated soil (Zhou et al., 2023). While chemical management of TR4 banana wilt is often quick and effective, it can lead to soil and aquatic environmental damage, and excessive usage may result in the development of resistance (Zhang et al., 2021a; Li et al., 2021b). In pursuit of sustainable agricultural practices, attention has shifted towards green and pollution-free biological agents. In recent years, endophytic bacteria have gained prominence due to their ability to enhance plant growth, manage soil-borne infections, and their environmentally friendly, safe, and non-toxic characteristics (Glick, 2012; Papik et al., 2020). Therefore, the selection of highly potent and broad-spectrum resistant bacteria is crucial for the development of effective biocontrol formulations.

*Bacillus endophyticus* is a potential biocontrol resource characterized by high resistance, broad-spectrum antimicrobial resistance and low pathogenicity to plants (Mon Myo et al., 2019; Fan et al., 2021). Furthermore, they have the capacity to generate a wide range of secondary metabolites that are capable of successfully impeding plant diseases and causing plant colonization, it encourages plant development (Ma et al., 2017). They are thought to be the best biocontrol agents for preventing diseases caused by soil-born pathogens like *Foc* (Fan et al., 2021). Banana wilt is now effectively controlled by a wide variety of biocontrol *bacilli*, such as *Bacillus amyloliquefaciens*, *Bacillus subtilis*, *Bacillus velezensis* and many others (Fan et al., 2021; He et al., 2021), all of which have better control effect on banana wilt. Therefore, the use of *Bacillus* to control banana wilt has a promising prospect. In this study, 11 strains of antagonistic bacteria were screened from 60 strains of smooth vetch (**Figure 1**; **Supplementary Figure S2**). Among which strain JSZ06 and its secondary metabolites exhibited strong inhibitory activity against *Foc* TR4 and 10 phytopathogenic fungi (**Figure 4**). After morphological and molecular biological, physiological, and biochemical characterization, strain JSZ06 was identified as *Bacillus siamensis* (**Figure 2**; **Supplementary Tables S1**, **S2**). Antibiotic drugs and their precursor substances produced by *Bacillus* spp. are widely used in agriculture, industry and medical industry (Sharon et al., 1954; Tiffin, 1958). The extracts isolated in this study by ethyl acetate and methanol showed effective antifungal activity, which was consistent with the *in vivo* inhibitory activity, and the effectiveness of the blockage was positively correlated to each extracts’ quantity. 200 mg/L of extract completely stopped the development of *Foc* TR4 hyphae (**Figures 5A, B**) and completely inhibited spore growth of *Foc* TR4 at concentrations equal to or greater than 8 × EC_50_ (**Supplementary Figure S5**). Inhibition of pathogen spore germination is important to protect crop and soil health (Chang et al., 2021; Fan et al., 2023). It reduces crop infestation caused by spore germination and the number of dormant spores in the soil and is an effective way to control banana wilt (Kammadavil Sahodaran et al., 2019; Chang et al., 2021). Similar findings showed *Bacillus* extracts could significantly inhibit *Foc* TR4 mycelial and sporulated growth (Ponnusamy et al., 2018; Shen et al., 2022).

Biofilm systems and organelles are crucial for preserving the cell shape and function of pathogenic fungi (Li et al., 2021b). In this research, a *Foc* TR4 mycelium treated with extracts of JSZ06 showed deformities, cross-linking, swelling, shortened internodes and increased branching, and degradation of cell walls, cell membranes and organelles (**Figures 5C, D**). By PCR amplification, it was detected that strain JSZ06 possessed 12 antimicrobial substance-related synthesized genes (**Supplementary Figure S3**, **Supplementary** **Table S4**), which may use gene expression to block the proliferation of *Foc* TR4 and produce antimicrobial compounds (Fan et al., 2021; Li et al., 2021a). Many investigations have shown that metabolites produced by antagonistic bacteria all lead to the loss of cellular integrity of pathogenic fungi (Chen et al., 2018; Wei et al., 2020; Li et al., 2021c; Zou et al., 2021), and the possible mechanism is to induce the activation of chitinase to hydrolyse the *Foc* TR4 cell wall (Zhang et al., 2021a), inhibit *Foc* TR4 biosynthesis and induce oxidative stress and apoptosis (Chen et al., 2018; Chen et al., 2020), which ultimately leads to the damage of the fungal outer wall membrane structure and the function of the internal organelles, the leakage of the contents, and the inhibition of the growth of phytopathogenic fungi. The identification of antimicrobial substance-related synthesis genes in the genome of the antagonist fungus JSZ06 supports this assumption. In conclusion, these findings further reveal the potential antagonistic mechanism of strain JSZ06 against *Foc* TR4.

In order to further reveal the mechanism, the antifungal properties of JSZ06 were preliminarily characterized using LC-MS coupling technique (**Supplementary Figures S10**, **11**, **Supplementary** **Table S6**). LC-MS plays an important role in the discovery of microbial metabolites (Caldeira et al., 2011). The extracts of strain JSZ06 contained a variety of antibacterial chemicals, the largest group being aminoglycoside antibiotics, which exhibits potent antibacterial activity as has been extensively shown (Khan et al., 2020; Wang et al., 2022). Zimmermann et al. (2016) reported that the synthesis of Neamine antibacterial amphiphilic AG enables the synthesis of new antifungal derivatives. It has also been found that Gentamicin and Hygromycin B can be used as antifungal drugs (Farzaneh, 2021), and that Hygromycin B binds to steroids on the fungal cell membranes, altering the membrane permeability and causing disruption of the fungus, and thereby actimg as a fungicide (Brodersen et al., 2000; Armillas Canseco et al., 2015). Additional tetracycline antibiotics include Sarecycline, Rolitetracycline, Minocycline, and Oxytetracycline, of which Oxytetracycline has been shown to inhibit the growth of fungal populations in the soil (Wang et al., 2017). Two amide antibiotics, Macrolactin A (7-O-Succinyl) with Cycloheximide, were also found, and Olishevska et al. (2019) discovered that 7-O-Succinyl macrolactin A, which *Bacillus amyloliquefaciens* was capable to generate, has an antifungal efficacy against both plant infections and damaged fungal cells (Kawai et al., 2023). Validamycin A and Actinonin were also found to be two types of natural antimicrobial agents. Actinonin as an antimicrobial agent has not yet been fully elucidated in terms of its mechanism of resistance to phytopathogenic fungi, but numerous investigations have shown possible mechanisms that can lead to inhibition of fungal growth through the inhibition of fungal protease activity and interference with fungal cell wall synthesis and metabolic pathways (Chen et al., 2000). Validamycin A, as a broad-spectrum antifungal pesticide, has been used in large quantities for the control of plant diseases, which can prevent fungus development and reproduction by inhibiting the synthesis of fungal wall polysaccharides, and interfering with fungal sugar metabolism and biosynthesis pathways (Wu et al., 2017; Bian et al., 2021). Some ester antibiotics, including Erythromycin C, Epothilone C and Surfactin, were also identified (Nayeem et al., 2009). Surfactin in particular is thought to have significant activity antagonistic to *Foc* (Vitullo et al., 2012), which can rupture fungal cell membranes, resulting in damage to the cell membrane leading to cell death and lysis (Fatin et al., 2017; Chen et al., 2022), thereby inhibiting spore germination and impeding the reproduction and infestation of the fungus (Romero et al., 2007). Surfactin additionally induces the upregulation of genes associated with plant defence and stimulates the synthesis of antimicrobial compounds in plants (Ongena and Jacques, 2008). Notably, the presence of surfactin in both the extracts of the strain identified by LC-MS and the gene cluster products predicted by the above mentioned antiSMASH further validates the above described inference that surfactin plays a substantial role in the antimicrobial effect exerted by strain JSZ06 (**Supplementary Figure S8**, **Supplementary Table S5**). However, there were also some substances that failed to match each other, likely as a result of the detection technique’s inability to detect all of strain JSZ06’s compounds (Li et al., 2021c). It has also been shown that the yield and number of antagonistic substances can be further increased by optimization of fermentation conditions (Duan et al., 2020; Zhang et al., 2021b) (**Supplementary Figure S4**). It is therefore assumed that all of these substances are important in supporting JSZ06’s wide-ranging antifungal action.

The pot inoculation test showed that the antagonist bacterium JSZ06 possesses a powerful biocontrol impact against banana wilt (**Figure 6**). In this study, GFP-*Foc* TR4 was employed to track the TR4 infection process in banana cultivars (He et al., 2021) (**Figure 7**). These findings demonstrated that TR4 may pierce the root epidermis, invade the xylem conduit to form mycelium, and expand and grow a mycelium in banana tissues. At the later stage of disease development, TR4 would form new spores in banana tissues, thus starting a new round of the infestation process. These occurrences generally concur with the findings of earlier research (Ploetz and Pegg, 1997; Ploetz, 2006; Li et al., 2011; Zhou et al., 2023). Prior studies on the TR4 infection pathway have focused on the invasion of plant bulbs (Dita et al., 2018; Zou et al., 2021; Li et al., 2021b). In our investigation, we noted that the TR4 mycelium entered the bulb through the vasculature from the root, and the amount of TR4 mycelium in the root system was significantly greater compared to the bulb. Meanwhile, the number of mycelia in banana roots and bulbs (JSZ06 + TR4) when JSZ06 was applied was noticeably less than that in the TR4 treatment (TR4), proving that strain JSZ06 can further prevent the pathogen from infecting banana.

In addition, the pot inoculation test greatly aided in the development of banana seedlings in terms of thick stems, plant height, leaf length, leaf width, as well as above-ground and below-ground fresh biomass (**Figure 8**). In addition to greatly reducing banana wilt, the antagonist bacteria promoted the development of banana plants (Fan et al., 2021). Further investigations into the mechanism of action suggest that it may be related to the creation of IAA, iron carrier, ACC deaminase, solubilized inorganic phosphorus as well as activated potassium by the antagonist bacterium JSZ06 (**Figure 3**). Biocontrol bacteria with production of iron carriers and IAA can promote plant growth and increase stress resistance (Hassan, 2017; Lü et al., 2023). Iron carriers not only compete for pathogenic iron and interact with pathogenic iron-associated enzymes, and thereby inhibiting growth-limiting pathogens, but also enhance plant health and productivity by improving the plant’s iron nutritional status (Compant, 2005; Shen et al., 2022). Bacteria with the ability to detoxify phosphorus and potassium have great potential in regulating plant growth (Rodríguez and Fraga, 1999; Vessey, 2003; Hassan, 2017), while ACC deaminase reduces ethylene in plants and improves rooting and tolerance for plant growth (Glick, 2014). Therefore, the antagonist bacteria JSZ06’s showed strong bacteriostatic and life-promoting properties, which may have many uses in agricultural production. It is worth noting that the promotion of endophytic strain JSZ06 may also be related to the finalization mechanism of the strain in plant roots (Sahu et al., 2023), to be followed up with further studies. Several studies have also found that plants recruit specific microorganisms from surrounding soil species to colonize the plant, become core microorganisms and perform specific functions (Lundberg et al., 2012; Bulgarelli et al., 2013), which further cinfirms the study of endophytes in smooth vetch.

## 5 Conclusion

The antagonist bacterium *Bacillus siamensis* JSZ06, along with its extracts, exhibited potent bacteriostatic effects against *Fusarium oxysporum* f. sp. cubense Tropical Race 4 (*Foc* TR4), effectively inhibiting mycelial development and spore germination of this pathogen. Treatments with extracts from the JSZ06 strain led to notable deformations of mycelial cells and the dissipation of internal structures. Consequently, all ten tested phytopathogenic fungi demonstrated susceptibility to the broad-spectrum suppressive activity of both the JSZ06 strain and its crude extracts. Strain JSZ06 was found to possess 12 pairs of bioprophylaxis-promoting related genes, which play a key role in the synthesis of antibiotics, as well as key growth hormone synthase genes. Further investigations into the growth-promoting abilities of the JSZ06 strain uncovered a significant potential, marked by the capacity for indole-3-acetic acid (IAA) production, iron chelation, ACC deaminase activity, and the solubilization of inorganic phosphorus and potassium. Moreover, the application of the fermentation broth derived from *Bacillus siamensis* JSZ06 notably enhanced the resistance of banana plants to *Foc* TR4 infection and stimulated the growth of banana seedlings. Twenty-seven primary inhibitory active substances were identified within the extracts of the JSZ06 strain. This study demonstrates that *Bacillus siamensis* JSZ06 can serve as an effective biocontrol agent against *Foc* TR4 infestation in bananas, while concurrently promoting banana growth.

## Data availability statement

The datasets presented in this study can be found in online repositories. The names of the repository/repositories and accession number(s) can be found in the article/**Supplementary Material**.

## Author contributions

YR: Formal analysis, Writing – original draft, Writing – review & editing. CN: Conceptualization, Data curation, Investigation, Software, Writing – original draft. AJ: Visualization, Writing – review & editing. HF: Methodology, Writing – original draft. LF: Writing – original draft, Project administration, Supervision. SZ: Supervision, Methodology, Resources, Writing – review & editing. SL: Methodology, Supervision, Writing – review & editing, Data curation. ZW: Data curation, Methodology, Supervision, Writing – review & editing, Conceptualization, Formal analysis, Funding acquisition, Investigation, Project administration, Resources, Software, Validation, Visualization, Writing – original draft.
