# Supplementary material for: A smooth vetch (Vicia villosa var.) strain endogenous to the broad-spectrum antagonist Bacillus siamensis JSZ06 alleviates banana wilt disease
Source: Front Plant Sci. 2024 Jun 4;15:1410197. doi: 10.3389/fpls.2024.1410197 (PMC11229777; doi:10.3389/fpls.2024.1410197)
Supplement: Supplementary file 1 [file DataSheet_1.docx]

Supplementary Material

# 1 Supplementary **Figure**s

Figure S1 Smooth vetch. **(A)** Flowers, **(B)** Leaves, **(C)** Stem, **(D)** Smooth vetch plant, **(E)** Root and **(F)** Seeds.

Figure S2 Analyzing endophytes' antifungal effects on *Foc* TR4. **(A)** The growth of *Foc* TR4 is inhibited by antagonists of 11 strains. **(B)** The growth of *Foc* TR4 is inhibited by antagonists of extracts from 11 strains. **(C)** Quantitative evaluation of several isolates' antifungal activity against *Foc* TR4. **(D)** Quantitative evaluation of various isolation extracts' antifungal activity against *Foc* TR4. At the significance threshold of *P* < 0.05, different lowercase letters indicate a significant difference.

Figure S3 PCR Detection of Antimicrobial Substance Synthesis Genes in *Bacillus siamensis* JSZ06.

Figure S4 Effects of different pH **(A)**, temperature **(B)**, rotational speed **(C)** and inoculum volume **(D)** on the growth and fermentation broth inhibitory activity of strain JSZ06. Different lowercase letters indicate a significant difference at the level of *P* < 0.05.

Figure S5 Influence of JSZ06 strain extracts on spore germination inhibition and morphological characterisation of *Foc* TR4. **(A)** Using 10% DMSO as a control, the 1 ×, 2 ×, 4 ×, and 8 × EC_50_ extracts were used to inhibit the germination of *Foc* TR4 spores. **(B)** Impact of extract treatments on *Foc* TR4 spore morphological features. At the significance threshold of *P* < 0.05, different lowercase letters indicate a significant difference.


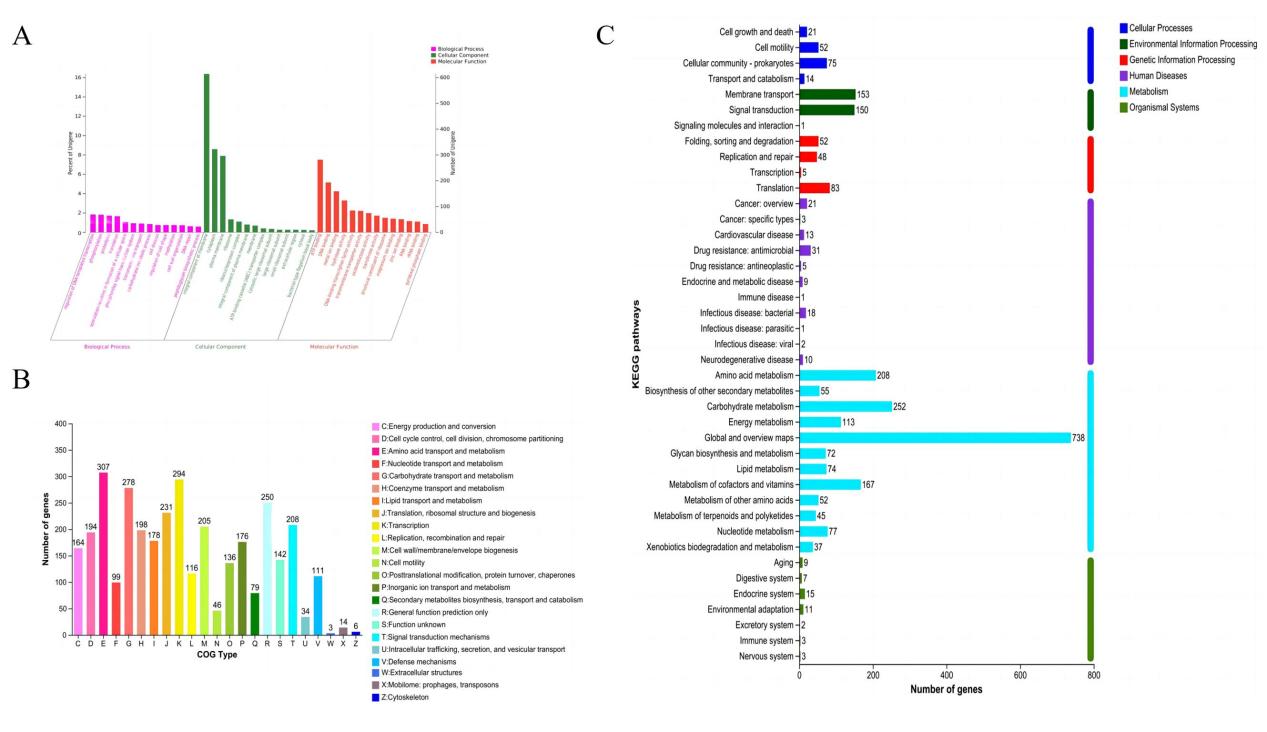
Figure S6 Genome annotation of strain JSZ06. **(A)** GO annotation of the genome of strain JSZ06. **(B)** COG annotation of the genome of strain JSZ06. **(C)** Pathway annotation of strain JSZ06 genome according to KEGG database.

Figure S7 Structural formula of the predicted products of the secondary metabolite synthesis gene cluster of strain JSZ06.

Figure S8 Annotation of the secondary metabolite synthesis gene cluster of strain JSZ06.

Figure S9 Total ion chromatogram. **(A)** Total ion chromatogram in the POS model. **(B)** Total ion chromatogram in the NEG model. x-axis represents retention time and y-axis represents the sum of the intensities of all ions in the MS.

Figure S10 Metabolite comparison and functional annotation of strain JSZ06. **(A)** Metabolite identification and annotation using the KEGG pathway classification. The KEGG pathway's level 2 terms are shown on the x-axis, while the number of metabolites is shown on the y-axis. **(B)** 16 HMDB superclasses were created from the metabolites found during secondary identification. The number of detected metabolites is shown on the y-axis, while the x-axis shows the HMDB superclasses.

Figure S11 Identified metabolites, categorized into 127 KEGG pathways. x-axis represents the top 50 KEGG pathways and y-axis represents the number of identified metabolites involved in the pathway.

# 2 Supplementary Tables

**TABLE S1 | Characteristics of strain JSZ06 on different solid culture media**

| **Culture medium** | **Pungent odor** | **Biofilm** | **Colony color** | **Colony Characteristics** | **Growth Conditions** | **Single Colony** | **Bacterial Lawn** |
| --- | --- | --- | --- | --- | --- | --- | --- |
| LB | Exist | Exist | Ash gray | Dry, more viscous, with many folds and bumps on the surface | +++ |  | 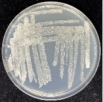 |
| PDA | Exist | None | Light yellow | Dry, easy to pick, pleated surface, chipped edges | ++ |  | 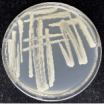 |
| R2A | None | None | Rice yellow | Dry, more viscous, with a pleated surface and chipped edges | + | 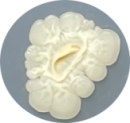 | 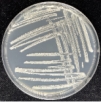 |
| NA | None | None | Yellowish white | Dry, much pleated in the center, much chipped at the edges | ++ | 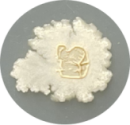 | 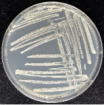 |
| CDA | None | None | Lemon yellow | Moist, transparent, inconsistent color, tree-rooted margins | + | 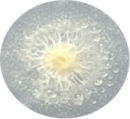 | 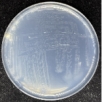 |
| TSA | Exist | None | Light yellow | Dry, more viscous, with a pleated surface and notched edges | +++ | 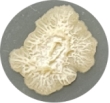 | 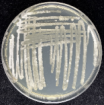 |
| Gause’s no.1 | None | None | Rice yellow | Moist, smoother, egg-white, margin notched | + | 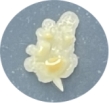 | 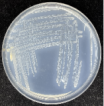 |

**TABLE S2 | Physiological and biochemical properties of antagonist bacteria JSZ06**

| **Measurement targets** | Result | **Measurement targets** | Result | **Measurement targets** | Result |
| --- | --- | --- | --- | --- | --- |
| Negative Control | - | Inosine | - | D-Glucuronic Acid | + |
| Dextrin | + | 1% Sodium Lactate | + | Glucuronamide | + |
| D-Maltose | + | Fusidic Acid | - | Mucic Acid | - |
| D-Trehalose | + | D-Serine | - | Quinic Acid | - |
| D-Cellobiose | + | D-Sorbitol | + | D-Saccharic Acid | - |
| Gentiobiose | + | D-Mannitol | + | Vancomycin | - |
| Sucrose | + | D-Arabitol | - | Tetrazolium Violet | + |
| D-Turanose | - | *myo*-inositol | + | Tetrazolium Blue | + |
| Stachyose | - | Glycerol | + | p-Hydroxyphenylaceticacid | - |
| Positive Control | + | D-Glucose-6-Phosphate | - | Methyl Pyruvate | - |
| pH6 | + | D-Fructose-6-Phosphate | + | D-Lactic Acid Methyl Ester | + |
| pH5 | + | D-Aspartic Acid | - | L-Lactic Acid | - |
| D-Raffinose | - | D-Serine | - | Citric Acid | + |
| α-D-Lactose | - | Troleandomycin | - | α-Keto-Glutaric Acid | - |
| D-Melibiose | - | Rifamycin SV | - | D-Malic Acid | - |
| β-Methyl-D-Glucoside | + | Minocycline | - | L-Malic Acid | + |
| D-Salicin | + | Gelatin | + | Bromo-Succinic Acid | + |
| N-Acetyl-D-Glucosamine | + | Glycyl-L-Prolin | - | Nalidixic Acid | - |
| N-Acetyl-β-Dmannosamine | - | L-Alanine | + | Lithium Chloride | + |
| N-Acetyl-D-Galactosamine | - | L-Arginine | + | Potassium Tellurite | + |
| N-Acetyl Neuraminic Acid | - | L-Aspartic Acid | + | Tween 40 | - |
| 1% NaCl | + | L-Glutamic Acid | + | γ-Amino-Butryric Acid | - |
| 4% NaCl | + | L-Histidine | - | α-Hydroxy-Butyric Acid | - |
| 8% NaCl | + | L-Pyroglutamic Acid | - | β-Hydroxy-D,L-Butyricacid | - |
| α-D-Glucose | + | L-Serine | - | α-Keto-Butyric Acid | - |
| D-Mannose | + | Lincomycin | - | Acetoacetic Acid | - |
| D-Fructose | + | Guanidine HCl | + | Propionic Acid | - |
| D-Galactose | - | Niaproof 4 | - | Acetic Acid | - |
| 3-Methyl glucose | - | Pectin | + | Formic Acid | + |
| D-Fucose | - | D-Galacturonic Acid | + | Aztreonam | + |
| L-Fucose | - | L-Galactonicacidlactone | + | Sodium Butyrate | - |
| L-Rhamnose | - | D-Gluconic Acid | - | Sodium Bromate | + |

+, Positive reaction; −, Negative reaction.

**TABLE S3 | The designed specific primers and housekeeping gene for RT-qPCR.**

| **Category** | **Metabolites** | **Synthesis Gene** | **Primers (5`-3`)** |
| --- | --- | --- | --- |
| Non-ribosomal peptide  synthetases (NRPS) | surfactin | *SrfAA* | AGCGTAAACGGCATTCAGGAG  TATGAGACGGCAGTGTTTCGG |
|  | YndJ | *yndJ* | CAGAGCGACAGCAATCACATC  ATTGCTCGGCAGGATCATACG |
|  | fengycin | *fenD* | CATTTTAACCAGTCCGTCATGC  TCTTTTTTGCAGACAAGGCGC |
|  | iturin | *ituC* | AACGAATACGGGCCTACAGAG  CTTCATGCTCTTATCCAGCACG |
|  | yngG | *yngG* | CAGAGCGACAGCAATCACATC  ATTGCTCGGCAGGATCATACG |
|  | bacillomycineD | *bamD* | ATTGGCGAAACGAAACATCTGC  AACATCTGATTGTGCTCACGTTC |
|  | bacillibactin | *dhb* | CAGTGAAATCGAGCCGATCC  TCTGAAACGGCTTTACAGCATG |
| Polytide synthetases  (PKS) | difficidin | *dfn* | TATCTCAATCGGATCGCCGAG  ATACGGTGCCTAATCCGGAAG |
|  | bacillaene | *bae* | TGTGCGGTCGTGTATGAACAG  AACGGTCTGTATAAATGCCGATG |
|  | macrolactin | *mln* | CTGATGAACTGATAACAACCGAG  ACGTGCCGAAACAACGATTGG |
|  | bacilysin | *bac* | TGAAGGGACAAGTAGTGAGTAC  AGGCACAATTGTGTATTCCAGC |
| Ribosomal peptide  Synthetases (RPS) | Subtilosin | *SboA* | GTCGCCGAAAAATCAAAAACGG  ACAAGCTCTATGCCGCACATG |

**TABLE S4 | The amplification characteristics of biocontrol marker genes and synthetic genes of strain JSZ06 in the test strains.**

| **Category** | **Category** | **Synthesis Gene** | **JSZ06** |
| --- | --- | --- | --- |
| **Non-ribosomal peptide**  **synthetases (NRPS)** | surfactin | *SrfAA* | + |
|  | YndJ | *yndJ* | + |
|  | fengycin | *fenD* | + |
|  | iturin | *ituC* | + |
|  | yngG | *yngG* | + |
|  | bacillomycine D | *bamD* | + |
|  | bacillibactin | *dhb* | + |
| **Polytide synthetases**  **(PKS)** | difficidin | *dfn* | + |
|  | bacillaene | *bae* | + |
|  | macrolactin | *mln* | + |
|  | bacilysin | *bac* | + |
| **Ribosomal peptide**  **Synthetases (RPS)** | Subtilosin | *SboA* | + |

“+” denotes a PCR result that is positive, while “-” denotes a negative result.

**TABLE S5 | Prediction and functional annotation of secondary metabolites of strain JSZ06 using online antiSMASH v4.2.0 software comparison**

| Region | Type | Location (bp) | Predicted compounds | Similarity | MIBiG accession | Gene number |
| --- | --- | --- | --- | --- | --- | --- |
| Cluster1 | NRPS | 323397-387374 | surfactin | 82% | BGC0000433 | 39 |
| Cluster2 | PKS-like | 924044-965288 | butirosin A,B | 7% | BGC0000693 | 40 |
| Cluster3 | Terpene | 1050167-1067575 | - | - | - | 22 |
| Cluster4 | Lanthipeptide-class-ii | 1188565-1217453 | - | - | - | 34 |
| Cluster5 | TransAT-PKS | 1389410-1477245 | macrolactin H | 100% | BGC0000181 | 43 |
| Cluster6 | TransAT-PKS, T3PKS, NRPS | 1696774-1797339 | bacillaene | 100% | BGC0001089 | 43 |
| Cluster7 | NRPS, TransAT-PKS, Betalactone | 1871081-2005381 | fengycin | 100% | BGC0001095 | 61 |
| Cluster8 | Terpene | 2034029-2055391 | - | - | - | 21 |
| Cluster9 | T3PKS | 2119230-2160330 | - | - | - | 49 |
| Cluster10 | TransAT-PKS | 2287706-2381498 | difficidin | 100% | BGC0000176 | 38 |
| Cluster11 | NRP-metallophore,NRPS, RiPP-like | 3006202-3057993 | bacillibactin | 100% | BGC0000309 | 44 |
| Cluster12 | Other | 3594292-3635710 | bacilysin | 100% | BGC0000309 | 41 |

**TABLE S****6 | The main antimicrobial compounds of strain JSZ06 were characterized by LC-MS.**

| Compound’s name | RT (min) | Area (%) | M/Z | MF | Mode | CAS ID | Activity | References |
| --- | --- | --- | --- | --- | --- | --- | --- | --- |
| Validamycin A | 2.1647 | 6.671 | 515.2455732 | C_20_H_35_NO_13_ | POS | 37248-47-8 | Bactericides | Zhang et al., 2022 |
| Neamine | 3.5606 | 5.379 | 287.1709511 | C_12_H_26_N_4_O_6_ | POS | 3947-65-7 | Antibacterial, Antitumor | Zimmermann et al., 2016 |
| Apramycin | 3.3967 | 6.345 | 578.2485965 | C_21_H_41_N_5_O_11_ | POS | 37321-09-8 | Antibiotics | Zheng et al., 2023 |
| Hygromycin B | 1.1763 | 6.562 | 566.1934248 | C_20_H_37_N_3_O_13_ | POS | 31282-04-9 | Antibiotics | Yun et al., 2023 |
| Daunorubicin | 2.044 | 5.493 | 545.2080425 | C_27_H_29_NO_10_ | POS | 20830-81-3 | Antibacterial and antitumor | Namazi et al., 2023 |
| Sarecycline | 2.0971 | 6.624 | 520.2243862 | C_24_H_29_N_3_O_8_ | POS | - | Antibiotics | Zhang et al., 2023 |
| Cephaeline | 4.9229 | 6.670 | 530.2970950 | C_28_H_38_N_2_O_4_ | POS | 483-17-0 | Antiviral | - |
| Rolitetracycline | 7.8795 | 5.436 | 264.6205775 | C_27_H_33_N_3_O_8_ | POS | 751-97-3 | Antibiotics | Tsai et al., 2023 |
| Surfactin | 6.1257 | 8.530 | 1036.6892580 | C_53_H_93_N_7_O_13_ | POS | 24730-31-2 | Bactericides | Fan et al., 2021 |
| Actinonin | 4.1213 | 4.610 | 427.2909233 | C_19_H_35_N_3_O_5_ | POS | 13434-13-4 | Antibacterial, Antitumor | Yoon et al., 2004 |
| Minocycline | 1.8589 | 7.047 | 490.2140058 | C_23_H_27_N_3_O_7_ | POS | 10118-90-8 | Antibiotics | Shan et al., 2002 |
| Erythromycin C | 4.7906 | 5.137 | 758.4084143 | C_36_H_65_NO_13_ | POS | 114-07-8 | Antibiotics | I O et al., 1985 |
| 7-O-Succinyl macrolactin A | 5.8571 | 5.812 | 520.2901199 | C_28_H_38_O_8_ | POS | - | Antibacterial, Antitumor | Jin et al., 2017 |
| Retaspimycin | 5.5068 | 5.606 | 651.3342896 | C_31_H_45_N_3_O_8_ | POS | - | Antibacterial, Antitumor | Wagner et al., 2013 |
| Gentamicin C | 3.4516 | 6.361 | 488.2498504 | C_19_H_39_N_5_O_7_ | POS | 11097-82-8 | Antibiotics | Copper et al., 1971 |
| Epothilone C | 5.6314 | 5.103 | 510.2924520 | C_26_H_39_NO_5_S | POS | - | Antibiotics | Nayeem et al., 2009 |
| Manumycin A | 5.6832 | 5.773 | 595.2686716 | C_31_H_38_N_2_O_7_ | NEG | 52665-74-4 | Antibiotics | Byung et al., 1996 |
| Dihydrostreptomycin | 5.5871 | 5.657 | 582.2788362 | C_21_H_41_N_7_O_12_ | NEG | 128-46-1 | Antibacterial | Lightbown and Jackson, 1956 |
| Novobiocin | 3.8503 | 5.501 | 633.2031827 | C_31_H_36_N_2_O_11_ | NEG | 303-81-1 | Antibacterial | Ys et al., 2015 |
| Oxytetracycline | 4.0661 | 6.633 | 495.1193151 | C_22_H_24_N_2_O_9_ | NEG | 79-57-2 | Antibacterial, Antitumor | Elia et al., 2014 |
| Aerobactin | 4.2974 | 6.033 | 545.2104130 | C_22_H_36_N_4_O_13_ | NEG | 545.210413 | Antibiotics | Choi et al., 2022 |
| Geneticin | 5.7709 | 5.562 | 517.2447362 | C_20_H_40_N_4_O_10_ | NEG | 49863-47-0 | Bactericides,  Antiviral | Varricchio et al., 2022 |
| 7-Aminocephalosporanic acid | 3.9862 | 5.038 | 271.0397054 | C_10_H_12_N_2_O_5_S | NEG | 957-68-6 | Bactericides | Ozcelik et al., 2016 |
| Cephamycin C | 3.6264 | 5.605 | 467.0876586 | C_16_H_22_N_4_O_9_S | NEG | 38429-35-5 | Antibiotics | Ünsaldı et al., 2021 |
| Neomycin | 5.7709 | 5.071 | 659.3109568 | C_23_H_46_N_6_O_13_ | NEG | 119-04-0 | Antimicrobial activity | Cangiano et al., 2023 |
| Ribostamycin | 4.2575 | 5.918 | 491.1787204 | C_17_H_34_N_4_O_10_ | NEG | 491.1787204 | Antibiotics | Kong et al., 2020 |
| Cycloheximide | 3.7545 | 5.396 | 318.1096063 | C_15_H_23_NO_4_ | NEG | 66-81-9 | Antibiotics | Kawai et al., 2023 |
